# A conserved virulence backbone and heterogeneous type III secretion effector profiles in historical canine otitis-associated *Pseudomonas aeruginosa* from Hungary

**DOI:** 10.3389/fvets.2026.1951261

**Published:** 2026-09-10

**Authors:** Mercédesz Adrienn Veres, Balázs Moravcsik, Patrik Mag, Eszter Kaszab, Enikő Fehér, Ákos Jerzsele, Ádám Kerek

**Affiliations:** 1 Department of Pharmacology and Toxicology, University of Veterinary Medicine Budapest, Budapest, Hungary; 2 National Laboratory of Infectious Animal Diseases, Antimicrobial Resistance, Veterinary Public Health and Food Chain Safety, University of Veterinary Medicine Budapest, Budapest, Hungary; 3 Department of Bioinformatics, One Health Institute, Faculty of Health Sciences, University of Debrecen, Debrecen, Hungary; 4 Department of Microbiology and Infectious Diseases, University of Veterinary Medicine Budapest, Budapest, Hungary; 5 National Laboratory of Virology, Szentágothai Research Centre, University of Pécs, Pécs, Hungary

**Keywords:** canine otitis externa, exos, ExoU, one health, *Pseudomonas aeruginosa*, type III secretion system, virulome, whole-genome sequencing

## Abstract

**Background:**

Canine otitis externa provides a chronic, inflamed and frequently antimicrobial-exposed niche in which *Pseudomonas aeruginosa* can persist through a coordinated repertoire of adhesion, biofilm, iron-acquisition, secretion and toxin systems.

**Methods:**

We characterized the virulence-associated genomic architecture of a historical Hungarian collection obtained from canine otitis cases in 2010 and 2017. Of the 70 isolate identifiers in the nominal range 70–139, 60 had corresponding long-read assemblies and linked VFDB screening records. One assembly was excluded after genome-based taxonomic validation showed that it did not meet the species-level criterion for *P. aeruginosa*.

**Results:**

Primary absence-sensitive analyses were restricted to 52 genome-confirmed *P. aeruginosa* assemblies meeting prespecified CheckM criteria (completeness ≥95%, contamination ≤5%), while seven additional genome-confirmed *P. aeruginosa* assemblies that did not meet the primary CheckM criteria were retained only for descriptive contextualization and were excluded from prevalence denominators and absence-sensitive analyses. The primary cohort contained 242 distinct virulence-associated genes, with a median of 230.5 genes per isolate. Of these, 217 (89.7%) were core-like, occurring in at least 95% of genomes. Alginate, type IV pilus, flagellar, siderophore, phenazine, quorum-sensing, type II, type III and type VI secretion backbones were broadly conserved. In contrast, type III effector content was heterogeneous: *exoT* occurred in 52/52 isolates, *exoY* in 50/52, *exoS* in 49/52 and *exoU* in 4/52; one isolate showed assembly-level co-detection of *exoS* and an *exoU*-homologous sequence. Whole-virulome composition did not differ detectably between 2010 and 2017 (square-root Jaccard PERMANOVA, R^2^ = 0.0134, *p* = 0.6083), and no individual gene remained associated with collection year after false-discovery-rate correction.

**Conclusion:**

These data define a conserved historical virulence-associated genomic backbone punctuated by isolate-level effector and surface-structure variation. The detected genes are interpreted as genomic homologues and repertoire features rather than as evidence of their expression or functional contribution to pathogenicity.

## 1 Introduction

Canine otitis externa is among the most frequent disorders encountered in small-animal practice, with population-level studies estimating that approximately 7–10% of dogs receive this diagnosis in primary care (1). The condition is not a single infectious entity but a multifactorial inflammatory syndrome in which primary disease, anatomical predisposition and perpetuating pathological changes alter the external ear canal and permit secondary microbial overgrowth (1, 2). Among bacterial agents, *Pseudomonas aeruginosa* is particularly important in chronic, recurrent and treatment-refractory disease because it combines environmental persistence, broad metabolic adaptability, intrinsic antimicrobial resistance, rapid acquisition of additional resistance and a strong capacity for biofilm-associated growth (2–8).

The clinical challenge posed by *P. aeruginosa* cannot be explained by antimicrobial resistance alone. Successful colonization and persistence require coordinated attachment, motility, acquisition of limiting nutrients, evasion or manipulation of host responses and damage to epithelial tissues. The organism therefore deploys a multilayered virulence repertoire that includes flagella and type IV pili, lipopolysaccharide, alginate and other extracellular polysaccharides, pyoverdine and pyochelin siderophores, quorum-sensing networks, phenazines, rhamnolipids, extracellular proteases and phospholipases, exotoxin A and multiple secretion systems (9–15). These determinants operate as an interconnected architecture rather than as independent markers: quorum sensing regulates secreted enzymes and exoproducts, biofilm matrices alter spatial organization and stress tolerance, siderophores support growth under iron limitation, and secretion systems mediate both host-cell manipulation and interbacterial competition (9, 16, 17). Quorum sensing is particularly relevant in this context because it coordinates population-level responses, biofilm-associated behavior, stress adaptation and collective responses to antimicrobial exposure, thereby linking virulence regulation with persistence and population dynamics (18).

Particular attention has focused on the type III secretion system (T3SS), which injects effectors directly into host cells. The canonical effectors ExoS, ExoT, ExoU and ExoY are not uniformly distributed across *P. aeruginosa* lineages and encode distinct pathogenic strategies (16, 19–21). ExoS has ADP-ribosyltransferase and GTPase-activating activities that disrupt cytoskeletal and signaling pathways, whereas ExoU is a potent phospholipase associated with rapid cytotoxicity in experimental and human clinical settings (16, 21). ExoT is widely conserved and interferes with cytoskeletal dynamics and phagocytosis, while ExoY is a host-activated nucleotidyl cyclase whose contribution depends on the wider effector context (16, 20). Although *exoS* and *exoU* are often mutually exclusive, genomic co-detection has occasionally been reported (7, 21, 22); however, assembly-level co-detection alone does not establish coding-sequence integrity, expression or functional co-expression.

Whole-genome sequencing has begun to clarify the population biology of canine otitis-associated *P. aeruginosa*. Recent European, Asian and national studies show that canine isolates are genetically diverse, span multiple phylogenetic backgrounds and sequence types, and generally do not form a host-restricted lineage (4–8, 22, 23). A multinational analysis found no canine-specific virulence profile and showed that virulence-gene complement did not define a single otitis-associated cluster, despite frequent strong biofilm formation (7). Comparable studies in other animal-associated bacterial populations have likewise shown that virulence-marker distributions can vary substantially among isolates and should therefore be interpreted within their host, epidemiological and genomic context rather than as uniform species-level signatures (24). A later Hungarian cohort collected in 2024 similarly displayed a broad conserved virulence background but heterogeneous T3SS effector content, including *exoU* in 15 of 70 isolates (22). These findings support an opportunistic model in which widely distributed environmental lineages exploit the inflamed ear rather than a model based on a unique canine-adapted pathotype.

Historical collections remain valuable because they provide a baseline against which contemporary populations can be interpreted. However, gene absence in draft genomes and database-derived virulomes—the predicted virulence-associated gene repertoire—is particularly vulnerable to assembly quality, allele divergence and threshold selection. Absence-sensitive analyses therefore require stringent genome-quality filtering, explicit reporting of identity and coverage thresholds and sensitivity analyses at alternative thresholds (25–34). The present study examined an independent historical collection of canine otitis-associated isolates obtained in Hungary in 2010 and 2017. This historical collection is distinct from the later Hungarian cohort sampled in 2024 and characterized in complementary virulence- and resistance-focused studies (22, 23). Our objectives were to define the conserved and accessory virulence architecture, resolve T3SS effector virulotypes, assess whether virulome composition differed between the two collection years, and quantify the sensitivity of selected findings to genome-quality and VFDB threshold choices.

## 2 Materials and methods

### 2.1 Study design and bacterial isolates

This retrospective genomic study used an archival strain collection maintained at the Department of Pharmacology and Toxicology, University of Veterinary Medicine Budapest. The nominal collection comprised isolate identifiers 70–139, corresponding to 70 bacterial entries obtained from canine otitis externa cases in Hungary during 2010 and 2017. Isolates had been collected during routine diagnostic investigations and cryopreserved before the present study was initiated. Only one clinical isolate per dog was included in the analytical collection, and no dog contributed more than one isolate. Accordingly, the dataset contained no repeated within-dog observations from bilateral ear sampling, repeat visits or recurrent episodes of otitis. No prospective sampling, intervention or procedure involving live animals was performed. Sequencing was performed within a wider laboratory sequencing programme, but isolate assignment, quality control and all analyses reported here were handled as a separate historical cohort.

**Figure 1:**
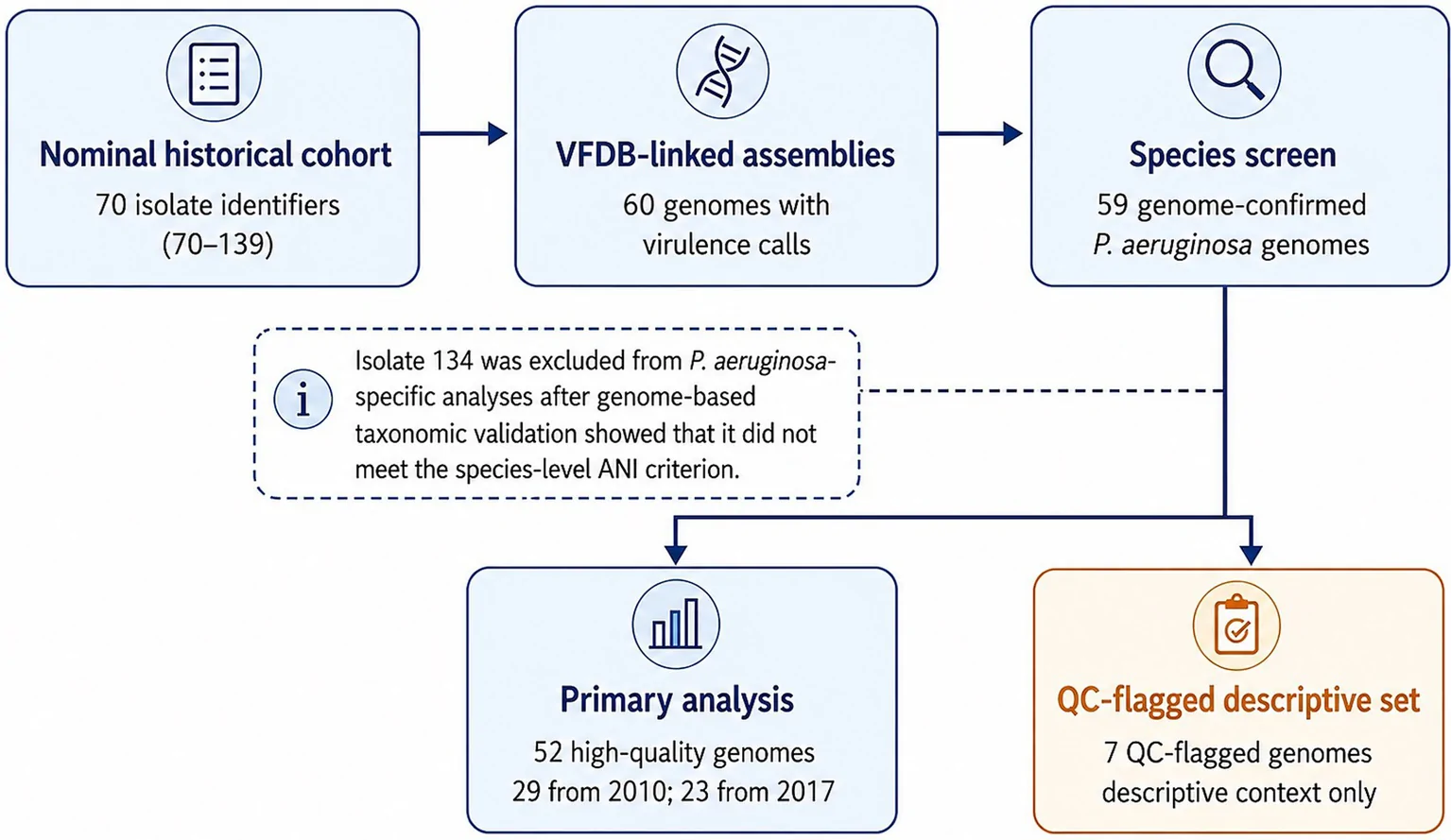
Cohort curation and quality-control flow for the historical canine otitis-associated *Pseudomonas* collection. The nominal identifier range contained 70 entries; 60 had linked VFDB-screened assemblies. Isolate 134 was excluded from *P. aeruginosa*-specific analyses after genome-based taxonomic validation showed that it did not meet the species-level ANI criterion. Primary absence-sensitive analyses used 52 high-quality genomes; seven QC-flagged genome-confirmed *P. aeruginosa* assemblies were excluded from prevalence denominators and retained only in the supplementary descriptive genome-topology visualization.

Virulence screening output was available for 60 assemblies within the nominal identifier range. For the remaining 10 nominal identifiers, no linked genome assembly and corresponding VFDB screening record were available in the archived analytical dataset. The historical records available for the present retrospective study did not permit the underlying reason for assembly unavailability to be reconstructed reliably; therefore, these entries were not included in genome-based analyses. Collection year was linked from the isolate register. Analyses were organized hierarchically to avoid treating low-quality assemblies as evidence of true gene absence. A genome-based species-confirmation screen was applied before genome-quality filtering, followed by a prespecified primary set used for prevalence and absence-sensitive analyses and a secondary QC-flagged set retained only for descriptive contextualization (Table 1; Figure 1).

**Table 1:** Cohort definition and quality-control structure.

Analytical stage	*n*	Definition/disposition
Nominal identifier range	70	Isolate identifiers 70–139 from the historical 2010/2017 strain bank.
VFDB-linked assemblies	60	Assemblies with virulence-screening records.
Genome-confirmed *P. aeruginosa* set	59	Assemblies confirmed as *P. aeruginosa* by genome-wide ANI after exclusion of isolate 134; 52 proceeded to the primary set and seven to the QC-flagged descriptive set.
Primary set	52	CheckM completeness ≥95% and contamination ≤5%; used for prevalence and absence-sensitive analyses.
QC-flagged descriptive set	7	Failed one or both primary CheckM criteria; excluded from prevalence denominators and absence-sensitive analyses and retained only in the supplementary descriptive genome-topology visualization.
Primary set, 2010	29	High-quality isolates linked to 2010.
Primary set, 2017	23	High-quality isolates linked to 2017.

Isolates originated from diagnostic ear-swab submissions from dogs diagnosed with otitis externa in Hungarian small-animal practices in 2010 or 2017. The archived isolates were provided through the veterinary diagnostic laboratory collection used for this retrospective study. Clinic-level identifiers and sufficiently detailed geographical metadata were not retained in the historical archive and were therefore unavailable for stratified analysis. Both year groups originated from the same general context of routine diagnostic submissions rather than from a prospectively standardized sampling programme. Consequently, although the overall sampling context was comparable, equivalence of the underlying clinic and source populations between 2010 and 2017 cannot be demonstrated retrospectively. Primary isolation was performed on cetrimide medium (Biolab Zrt., Budapest, Hungary) using colony morphology and characteristic pigment production. Archived isolates were revived and re-identified by matrix-assisted laser desorption/ionization time-of-flight mass spectrometry (MALDI-TOF MS) using a Microflex LT instrument and the MALDI Biotyper system (Bruker Daltonics, Bremen, Germany) equipped with MBT Compass software v4.1 before genomic analysis using the 2026 reference library. Only isolates identified as *P. aeruginosa* with a MALDI Biotyper log(score) ≥ 2.0 were accepted as species-level identifications and included in the archived *P. aeruginosa* collection.

### 2.2 DNA extraction, long-read sequencing and assembly

Genomic DNA was extracted from pure bacterial cultures using the Quick-DNA Fungal/Bacterial Miniprep Kit (Zymo Research, Irvine, CA, United States) according to the manufacturer’s instructions. Native barcoded libraries were prepared using the Oxford Nanopore Technologies Native Barcoding Kit 24 V14 (SQK-NBD114.24) and sequenced on R10.4.1 MinION flow cells with a MinION Mk1B device. Base calling was performed with Dorado v7.6.8 through MinKNOW v24.11.10 using the super-accuracy model. Adapter and lambda-control sequences were removed with NanoLyse v1.2.1; NanoFilt v2.8.0 was used to trim 50 nucleotides from each read end and remove reads with mean quality below 8 or length below 500 bp. Read-quality summaries were generated with NanoPlot v1.43.0, as implemented in NanoPack (28).

*De novo* assemblies were generated with Flye v2.9.5-b1801 (29). Long reads were aligned back to the assemblies with minimap2 v2.28 (30), corrected with Racon v1.5.0 (31), and subsequently polished with Medaka v2.0.1 using the r1041_e82_400bps_sup_v4.2.0 model. Assembly statistics were evaluated using QUAST v5.0.2 (32). Per-isolate assembly length, contig count, assembly N50, CheckM completeness and contamination, and BUSCO completeness metrics are reported in Supplementary Table S1. Although NanoPlot was used for read-level quality assessment during the original sequencing workflow, the corresponding per-isolate summary outputs were not retained with the historical analytical dataset; consequently, read N50 and read-depth estimates could not be reported retrospectively without reprocessing the original reads. No assembly was classified as a formally closed chromosome because circularization and chromosome closure were not independently validated.

### 2.3 Genome quality and cohort definition

Assembly completeness and contamination were assessed using BUSCO v5.8.2 in auto-lineage mode (33) and CheckM v1.2.4 (34). Assemblies were considered high quality for the primary absence-sensitive analysis when CheckM completeness was at least 95% and contamination did not exceed 5%. Seven genome-confirmed *P. aeruginosa* assemblies failed one or both CheckM criteria and were therefore excluded from prevalence denominators and all absence-sensitive analyses. These QC-flagged genomes were retained only for descriptive placement in the supplementary core-genome visualization (Supplementary Figure S4); their positive VFDB calls were not used as a formal sensitivity analysis and no biological conclusion was tested on the basis of this secondary set.

Because isolate 134 showed marked genomic discordance with the remainder of the cohort, formal genome-based taxonomic validation was performed before final cohort definition. Average nucleotide identity was calculated using FastANI against the canonical *Pseudomonas aeruginosa* reference genomes PAO1 (GCF_000006765.1), PA14 (GCF_000014625.1) and LESB58 (GCF_000026645.1). The resulting ANI estimates were 80.24, 80.34 and 81.09%, respectively, all far below the conventional 95% species boundary for *P. aeruginosa*. The corresponding FastANI alignment fractions were 820/2000 fragments (41.0%) for PAO1, 828/2000 (41.4%) for PA14 and 862/2000 (43.1%) for LESB58. Because these ANI estimates lie near the lower operating range of the fragment-mapping approach, they were interpreted conservatively as evidence of marked genomic divergence from canonical *P. aeruginosa* references rather than as precise estimates of interspecies relatedness. Because the purpose of these comparisons was solely to determine whether isolate 134 could be retained within the *P. aeruginosa* analytical cohort, and because the low-identity, partial-fragment matches were not considered sufficient for a robust alternative species assignment, no alternative taxonomic identity was inferred. To place genome inclusion and exclusion on the same taxonomic footing, the FastANI analysis was subsequently extended to all 59 retained assemblies using PAO1 (GCF_000006765.1) as the reference genome. All 59 retained assemblies exceeded the conventional 95% ANI species boundary for *P. aeruginosa*, providing genome-based confirmation of their classification as *P. aeruginosa*. Isolate 134 was therefore treated as taxonomically discordant and excluded from the analytical cohort and from all *P. aeruginosa*-specific analyses.

### 2.4 Virulence-factor detection and functional organization

Genome assemblies were screened using ABRicate v1.4.0 against the Virulence Factor Database (VFDB; database accessed 2 April 2026) (25–27). The primary call set required at least 80% nucleotide identity and at least 80% reference-sequence coverage. Multiple hits to the same gene within an isolate were collapsed to a single presence call, retaining the highest-identity/highest-coverage match for descriptive summaries. A stricter joint threshold of 90% nucleotide identity and 90% reference-sequence coverage was used as a sensitivity analysis for selected genes. Because identity and coverage were increased simultaneously, this analysis was designed to assess sensitivity to overall call-stringency rather than to distinguish the independent effects of nucleotide identity and reference coverage. Because VFDB is reference dependent, missing calls were interpreted as failure to meet the stated homology criteria rather than definitive biological deletion, particularly for allele-diverse loci.

VFDB factor labels were mapped into 12 biologically interpretable modules: alginate and biofilm matrix; type IV pili and adhesion; flagellar motility; lipopolysaccharide; iron acquisition; phenazine and pyocyanin; quorum sensing and rhamnolipid; type II secretion; type III secretion apparatus; type III secretion effectors; type VI secretion; and secreted toxins and enzymes. Module completeness for each isolate was calculated as the proportion of the cohort-defined union of genes in that module detected in the isolate. Isolate-level module-completeness patterns were visualized as a heatmap (Supplementary Figure S3). Virulence genes were categorized as core-like (present in at least 95% of primary genomes), soft core (90 to <95%), shell (15 to <90%) or cloud (<15%). These categories describe prevalence within this cohort and are not intended as species-wide pangenome definitions.

### 2.5 Type III secretion effector virulotypes and supplementary core-genome visualization

T3SS effector profiles were defined from *exoS*, *exoU*, *exoT* and *exoY* calls. For the principal comparison, isolates were classified as *exoS*+/*exoU*−, *exoU*+/*exoS*−, *exoS*+/*exoU*+ or *exoS*−/ *exoU*−. Full four-effector profiles for all 52 high-quality genomes were retained in Supplementary Table S6. The isolate showing simultaneous *exoS* and *exoU* calls was subjected to additional sequence-level validation against the canonical PAO1 *exoS* (PA3841) and PA14 *exoU* (PA14_51530) loci. Reference coverage, nucleotide identity, contig localization and coding-sequence integrity were examined directly in the assembled genome. No independent read-level, PCR or Sanger confirmation of single-nucleotide sequence variation at the *exoU*-homologous locus was available. Accordingly, any apparent single-base indel observed in the long-read-only assembly was treated as unverified and potentially attributable to residual sequencing or assembly error. Effector calls were therefore interpreted strictly as genomic homology evidence and not as confirmation of intact coding sequence or functional protein production.

Genome assemblies were annotated using Prokka v1.14.6, and the resulting GFF3 files were used as input to Panaroo v1.8.0 (35). Maximum-likelihood inference was performed in IQ-TREE v3.0.1 (36) using the fast tree-search option and automatic model selection (−fast -m MFP); ModelFinder (37) selected GTR + F + I + R5 according to the Bayesian information criterion. The original phylogenetic analysis contained 61 assemblies. For visualization of the historical study cohort, isolate 134 and the non-cohort isolate 146 were pruned from the resulting topology, leaving 59 historical assemblies. No bootstrap or SH-aLRT resampling was performed. The resulting topology was visualized and annotated in the Interactive Tree of Life (iTOL) v6 (38) with collection year, genome-quality status, T3SS genomic profile and selected virulence-gene calls. Because the topology lacked branch-support resampling and external comparator genomes, it was retained only as a supplementary descriptive visualization of the historical cohort. It was not used for supported clade definition, lineage assignment, temporal population-structure inference, comparative cross-host phylogenomics, transmission inference, or any principal biological conclusion of the study.

### 2.6 Statistical analysis

Analyses were performed in Python 3.13 using NumPy and SciPy. Virulence-gene burden and module-level gene representation were summarized by medians and interquartile ranges. Differences between 2010 and 2017 were assessed using two-sided Mann–Whitney U tests for continuous or proportional module-level outcomes and Fisher exact tests for individual gene prevalence. Benjamini-Hochberg correction was applied separately to aggregate/module tests and gene-level tests; q < 0.05 was considered statistically significant. Effect direction and exact P and q values are reported rather than relying on significance labels alone.

Whole-virulome dissimilarity was calculated from the binary gene-presence matrix using Jaccard dissimilarity. To ensure consistency among ordination and inferential analyses and to address the non-Euclidean component of the untransformed Jaccard matrix, an element-wise square-root transformation was applied to the Jaccard dissimilarities. The same square-root-transformed Jaccard matrix was used for principal coordinates analysis (PCoA), PERMDISP and PERMANOVA. PCoA was performed by eigendecomposition of the double-centered squared-distance matrix, and the eigenvalue spectrum was inspected for negative eigenvalues. Homogeneity of multivariate dispersion between collection years was assessed using centroid-based PERMDISP with 9,999 permutations. The collection-year effect was tested using a one-way PERMANOVA with 9,999 unrestricted permutations of collection-year labels. A random seed of 20,260,711 was used for permutation analyses. Results are reported as F and permutation *p* values for PERMDISP and as pseudo-*F*, *R*^2^ and permutation *p* values for PERMANOVA.

## 3 Results

### 3.1 Cohort curation and genome quality

Of the 70 nominal isolate identifiers, 60 had linked genome assemblies and VFDB-screening records available for retrospective analysis; the remaining 10 identifiers lacked linked genomic data and were therefore not evaluable in the genome-based analyses. Genome-based taxonomic validation showed that isolate 134 was markedly inconsistent with *Pseudomonas aeruginosa*, with FastANI estimates of 80.24% against PAO1, 80.34% against PA14 and 81.09% against LESB58, all well below the conventional species-level boundary. The corresponding alignment fractions were 820/2,000 (41.0%), 828/2,000 (41.4%) and 862/2,000 (43.1%) fragments, respectively. Given the low ANI range, these values were interpreted as evidence of marked genomic divergence from the three canonical *P. aeruginosa* references rather than as precise measures of relatedness to an alternative species. No alternative species assignment was inferred. Isolate 134 was therefore excluded before *P. aeruginosa*-specific analysis, leaving 59 historical genomes in the analytical genome-confirmed *P. aeruginosa* set. Genome-based species confirmation was additionally extended to all 59 retained assemblies using PAO1 as the FastANI reference; every retained assembly exceeded the conventional 95% ANI species boundary for *P. aeruginosa*. Detailed per-isolate assembly, genome-quality and PAO1 ANI metrics, including assembly length, contig count, assembly N50, CheckM completeness and contamination, and BUSCO results, are provided in Supplementary Table S1. None of the assemblies was designated as a formally closed chromosome.

### 3.2 The historical cohort carried a dense, predominantly core-like virulence repertoire

**Figure 2:**
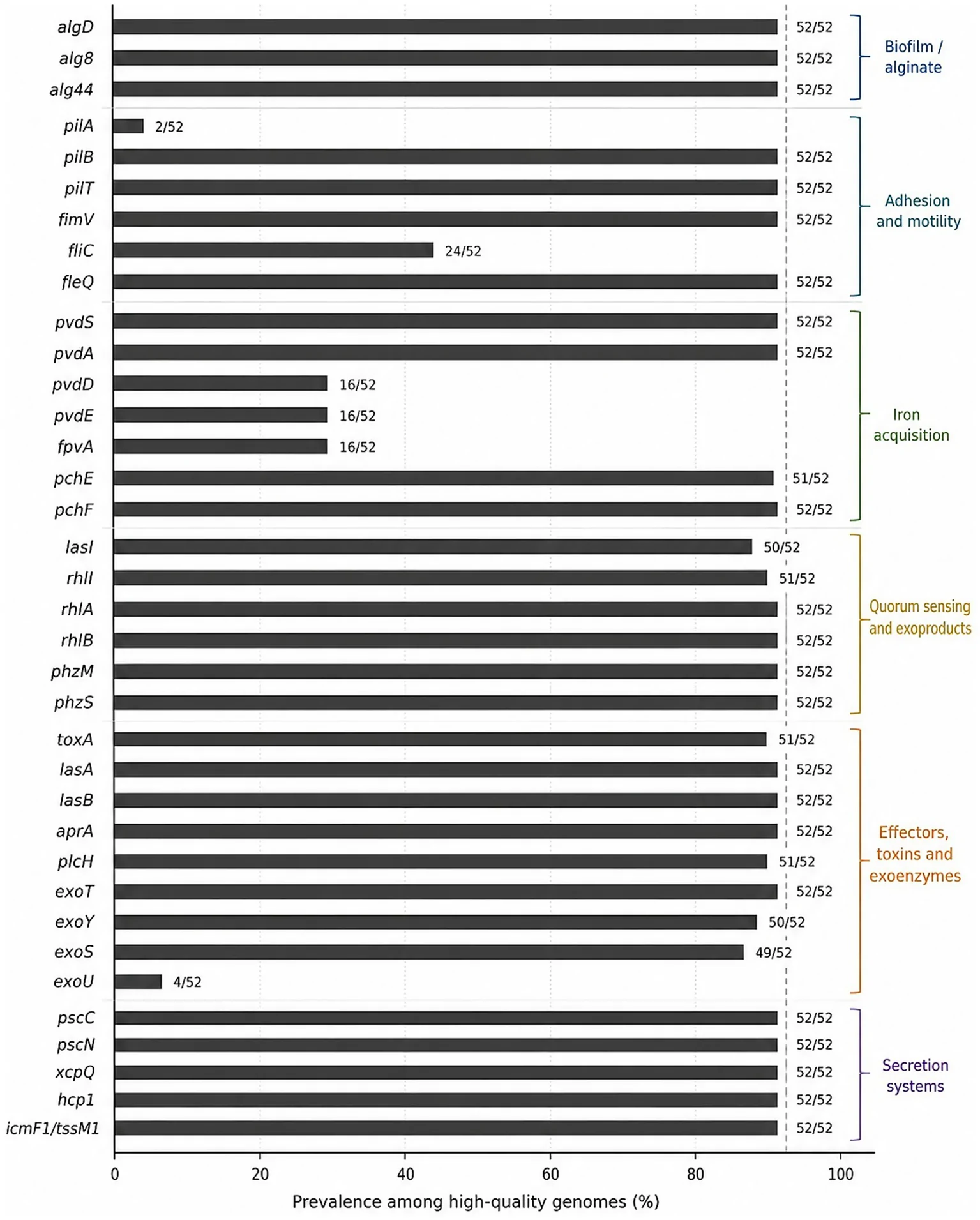
Prevalence of selected virulence-associated genes among the 52 high-quality *Pseudomonas aeruginosa* genomes. Bars show the percentage of primary genomes with a VFDB call meeting at least 80% nucleotide identity and 80% reference coverage. Numbers at bar ends indicate positive genomes/52. The dashed line marks the 95% core-like threshold.

The 52 high-quality genomes contained 242 distinct VFDB-associated genes at the primary threshold (Supplementary Table S2). The median isolate carried 230.5 genes (interquartile range 226.75–232.0; range 218–240). Of the cohort-wide repertoire, 217 genes were core-like, one was soft core, 22 were shell and two were cloud genes (Supplementary Figure S1). Thus, 90.1% of all detected genes occurred in at least 90% of high-quality genomes, defining a highly conserved virulence backbone despite isolate-level variation (Figure 2).

The core-like component included complete or near-complete alginate biosynthesis and regulation, type II and type III secretion apparatuses, HSI-I/HSI-1 type VI secretion components, phenazine and pyocyanin pathways, rhamnolipid biosynthesis, major extracellular proteases and most pyochelin-associated genes. The cohort-level density of these calls indicates that genetic determinants associated with attachment, matrix production, nutrient acquisition, secreted enzymes and interbacterial competition were broadly represented across the collection. Functional activity of these systems was not assessed in the present study.

### 3.3 Biofilm, adhesion, motility and iron-acquisition systems

**Figure 3:**
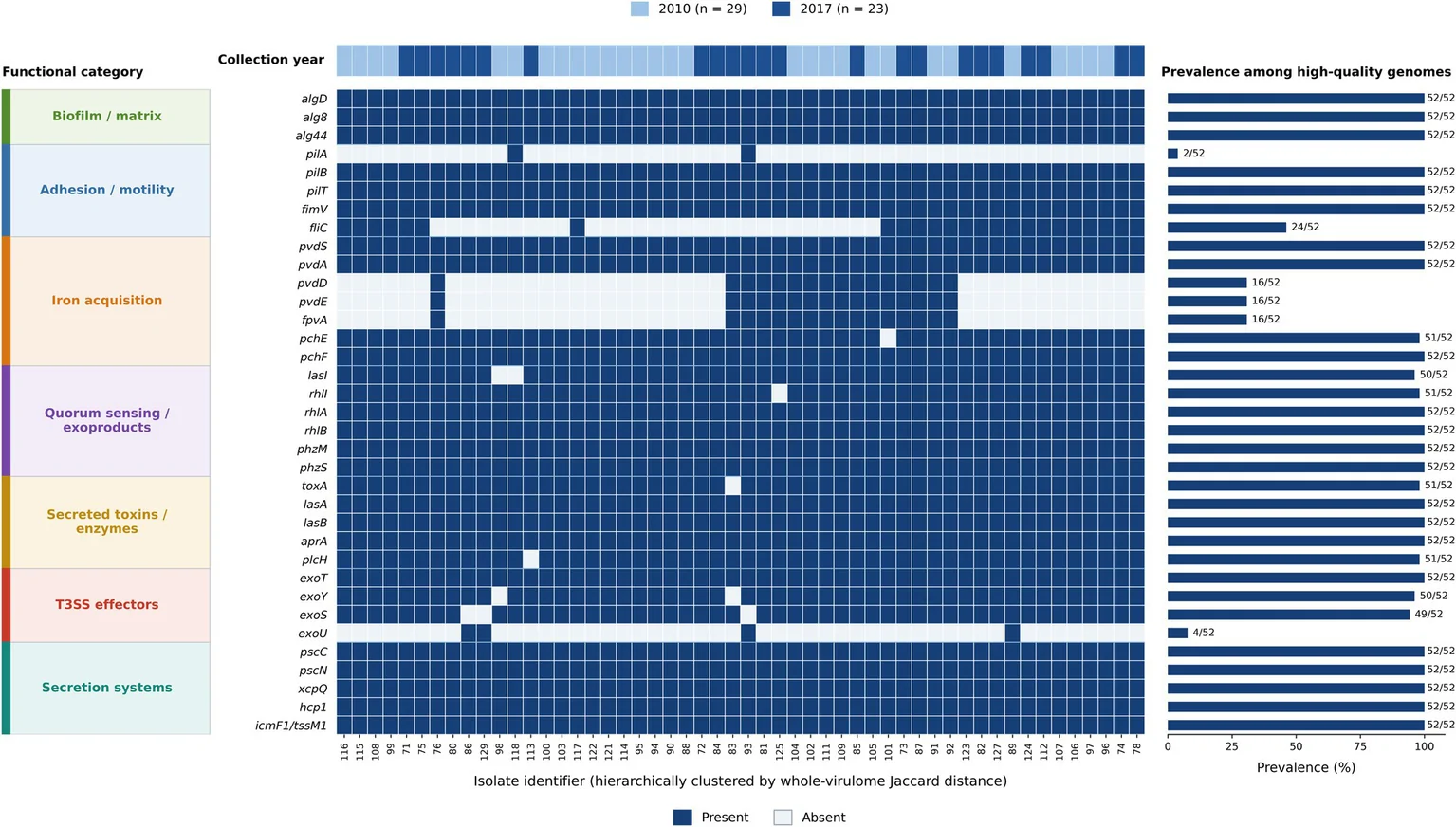
Selected virulence-gene presence/absence matrix for the 52 high-quality genomes. Isolates were ordered by average-linkage hierarchical clustering of whole-virulome Jaccard distances; genes were arranged by functional category. Dark blue denotes genomic detection at ≥80% nucleotide identity and ≥80% reference coverage, whereas pale blue denotes no call meeting both thresholds. The upper strip indicates collection year (light blue, 2010; dark blue, 2017). Apparent absence in allele-diverse loci should be interpreted as reference-threshold non-detection rather than confirmed functional deletion.

Canonical alginate-associated genes, including *algD*, *alg8* and *alg44*, were detected in all 52 high-quality genomes. The type IV pilus assembly and retraction backbone was likewise strongly conserved: *pilB*, *pilT* and *fimV* occurred in every primary genome. In contrast, the reference *pilA* call was detected in only 2/52 isolates, whereas most other pilus genes remained common. This pattern is more consistent with allelic divergence of the major pilin reference sequence than with wholesale loss of type IV pili, particularly because the broader pilus-associated gene repertoire was retained and the stricter threshold further reduced rather than rescued the *pilA* call. The flagellar regulator *fleQ* was universal, while the reference *fliC* allele was detected in 24/52 genomes (46.2%), again indicating locus-level sequence diversity within an otherwise conserved motility system (Figure 3).

Iron-acquisition capacity was similarly distributed across conserved and variable components. *pvdS* and *pvdA* were detected in all primary genomes, and *pchF* was universal; *pchE* occurred in 51/52. By contrast, the specific VFDB reference calls for *pvdD*, *pvdE* and *fpvA* were each detected in 16/52 genomes. The shared prevalence pattern of these pyoverdine-associated loci and their preservation under the stricter threshold for the positive subset suggest lineage-associated allele configurations rather than random assembly loss. Because siderophore loci are sequence-diverse, these prevalence values should be interpreted as reference-homology groups, not as direct measures of functional siderophore deficiency.

### 3.4 Quorum sensing, phenazines and secreted tissue-damaging factors were widely conserved

The quorum-sensing synthase genes *lasI* and *rhlI* were detected in 50/52 (96.2%) and 51/52 (98.1%) genomes, respectively, while *rhlA*, *rhlB*, *phzM* and *phzS* occurred in all isolates. The available VFDB output did not support a reliable absence analysis for every regulatory receptor and pathway component; accordingly, absence of uncalled regulators was not inferred. The observed pattern indicates widespread genomic representation of loci associated with acyl-homoserine-lactone, rhamnolipid and phenazine/pyocyanin biosynthesis.

Secreted toxins and enzymes were also nearly ubiquitous. *lasA*, *lasB* and *aprA* were present in 52/52 genomes, whereas *toxA* and *plcH* were present in 51/52. The complete *xcp* type II secretion backbone, represented by universal *xcpQ* and associated components, provides the secretion machinery required for multiple extracellular products. These results indicate that differences among historical strains were not driven by broad loss of the major proteolytic and cytotoxic repertoire.

### 3.5 T3SS apparatus conservation contrasted with effector heterogeneity

**Figure 4:**
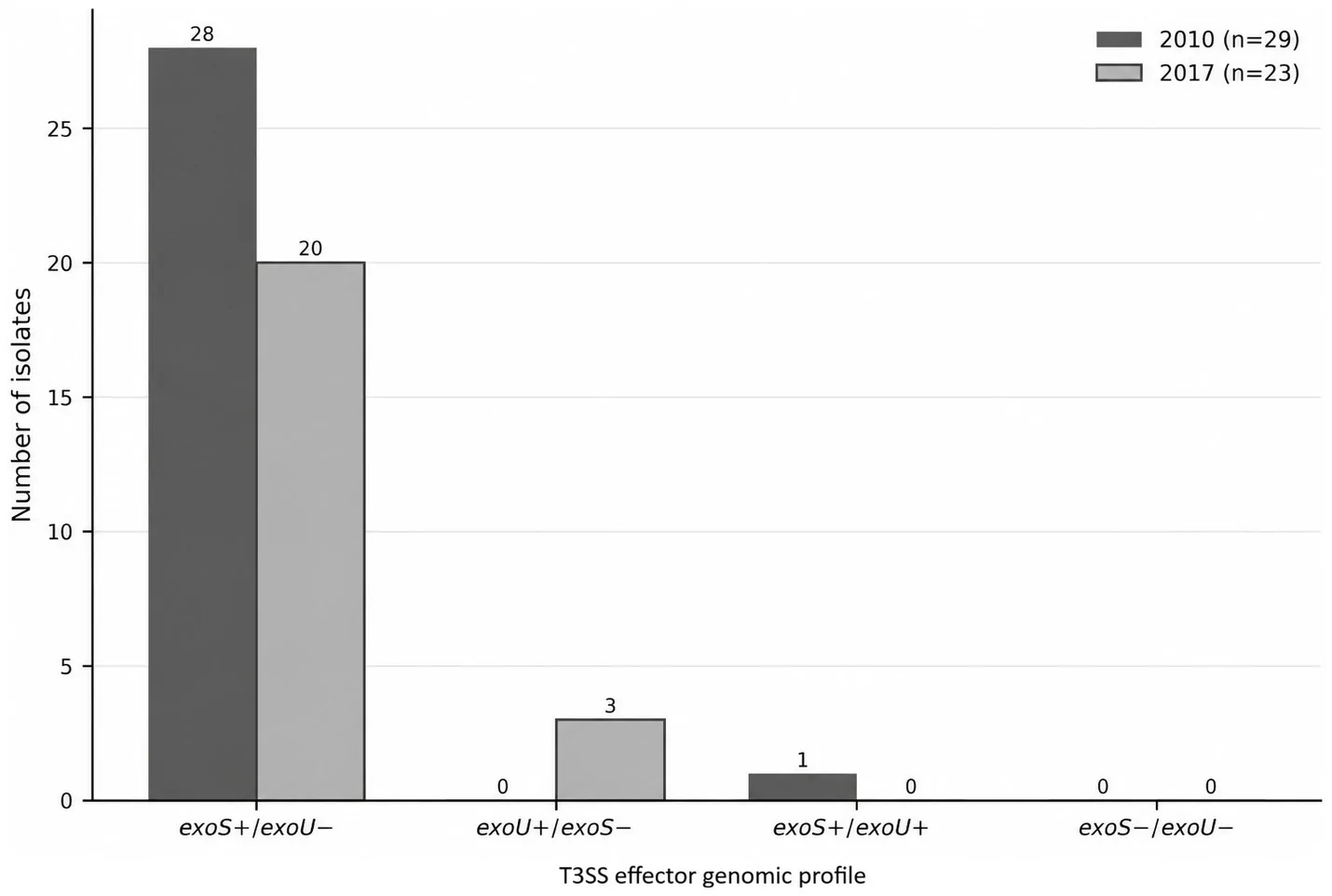
Distribution of principal type III secretion effector genomic profiles by collection year. Profiles were defined from VFDB-based genomic detection of *exoS* and *exoU* in the 52 high-quality genomes; *exoT* was detected in all isolates and *exoY* in all but two. One 2010 isolate showed assembly-level genomic co-detection of *exoS* and an *exoU*-homologous sequence. Assembly-level sequence inspection supported two distinct homologous loci, but no independent read-level, PCR or Sanger confirmation was available; therefore, coding-sequence integrity and functional co-expression of the corresponding effector proteins are not inferred.

Genes encoding the T3SS apparatus, including *pscC* and *pscN*, were detected in all primary genomes. Effector prevalence was less uniform: *exoT* was present in 52/52 (100%), *exoY* in 50/52 (96.2%), *exoS* in 49/52 (94.2%) and *exoU* in 4/52 (7.7%) (Table 2; Figure 3). Forty-eight isolates were *exoS*+/*exoU*−, three were *exoU*+/*exoS*−, and one 2010 isolate (isolate 89) showed assembly-level co-detection of *exoS* and *exoU* homologues (Figure 4; Table 3; Supplementary Table S6). Assembly-level inspection of isolate 89 showed full-length alignment of both loci. The *exoS* sequence showed 100% reference coverage and 99.49% nucleotide identity to PAO1 *exoS*. The *exoU*-homologous sequence covered 100% of the PA14 *exoU* reference, while the assembled sequence contained an apparent single-nucleotide insertion relative to the reference. In the assembly, this difference would alter the predicted reading frame; however, because no independent read-level or Sanger confirmation was available, the apparent insertion and resulting frameshift remain unverified and may represent residual long-read sequencing or assembly error. The *exoU* call was therefore interpreted only as evidence of an *exoU*-homologous genomic sequence and not as evidence either for or against production of an intact functional ExoU effector. The two homologous loci occurred on separate assembly contigs.

**Table 2:** Prevalence of selected virulence-associated genes in the 52 high-quality genomes.

Functional group	Gene	Present	Prevalence (%)	Median identity (%)
Biofilm/matrix	*algD*	52/52	100.0	100.0
Biofilm/matrix	*alg44*	52/52	100.0	99.7
Adhesion/motility	*pilB*	52/52	100.0	88.5
Adhesion/motility	*pilT*	52/52	100.0	99.7
Adhesion/motility	*fimV*	52/52	100.0	99.3
Adhesion/motility	*fliC*	24/52	46.2	99.9
Iron acquisition	*pvdS*	52/52	100.0	99.5
Iron acquisition	*pvdA*	52/52	100.0	99.0
Iron acquisition	*fpvA*	16/52	30.8	99.8
Iron acquisition	*pchE*	51/52	98.1	99.5
Quorum sensing/exoproducts	*lasI*	50/52	96.2	100.0
Quorum sensing/exoproducts	*rhlI*	51/52	98.1	98.5
Quorum sensing/exoproducts	*rhlA*	52/52	100.0	99.5
Quorum sensing/exoproducts	*phzM*	52/52	100.0	99.7
Secreted toxins/enzymes	*toxA*	51/52	98.1	99.3
Secreted toxins/enzymes	*lasA*	52/52	100.0	99.3
Secreted toxins/enzymes	*lasB*	52/52	100.0	99.7
Secreted toxins/enzymes	*aprA*	52/52	100.0	99.6
Secreted toxins/enzymes	*plcH*	51/52	98.1	99.7
T3SS effectors	*exoT*	52/52	100.0	99.1
T3SS effectors	*exoY*	50/52	96.2	99.6
T3SS effectors	*exoS*	49/52	94.2	99.6
T3SS effectors	*exoU*	4/52	7.7	99.9
Secretion systems	*pscC*	52/52	100.0	99.7
Secretion systems	*xcpQ*	52/52	100.0	99.6
Secretion systems	*hcp1*	52/52	100.0	100.0
Secretion systems	*icmF1/tssM1*	52/52	100.0	99.5

Identity values summarize the highest-scoring call per gene per isolate among positive genomes. Complete gene-level prevalence, median identity and median coverage data are provided in Supplementary Table S2.

**Table 3:** Principal T3SS effector virulotypes by collection year.

Virulotype	2010, *n* (%)	2017, *n* (%)	Total, *n* (%)
*exoS+/exoU−*	28 (96.6)	20 (87.0)	48 (92.3)
*exoU+/exoS−*	0 (0.0)	3 (13.0)	3 (5.8)
*exoS+/exoU+*	1 (3.4)	0 (0.0)	1 (1.9)
*exoS-/exoU−*	0 (0.0)	0 (0.0)	0 (0.0)

The exoS+/exoU+ category denotes threshold-based genomic co-detection. In isolate 89, assembly-level sequence inspection identified both homologues. The assembled exoU-homologous sequence contained an apparent single-nucleotide insertion that would alter the predicted reading frame; however, this indel was not independently confirmed and may represent residual long-read sequencing or assembly error. Neither intact exoU coding sequence nor functional ExoU production is therefore inferred.

The distribution differed descriptively between years: all 29 isolates from 2010 carried *exoS*, whereas 20/23 isolates from 2017 were *exoS*-positive; the three *exoS*-negative 2017 isolates carried *exoU*. However, the year association for *exoS* did not remain significant after correction across the full gene set (unadjusted *p* = 0.080; *q* = 1.000), and the difference in *exoU* prevalence was also non-significant (*p* = 0.310; *q* = 1.000).

### 3.6 Supplementary core-genome visualization

For completeness, the previously generated core-genome topology of the historical cohort is provided as Supplementary Figure S4. No bootstrap or SH-aLRT resampling was performed in the original analysis. Because branch support was therefore unavailable, the topology was not used to infer supported relationships among isolates. It was retained only as a supplementary descriptive visualization of the historical cohort and was not used for clade definition, lineage assignment, temporal population-structure inference, comparative cross-host phylogenomics, transmission inference, or any principal biological conclusion of the study.

### 3.7 No detectable whole-virulome shift between 2010 and 2017

**Figure 5:**
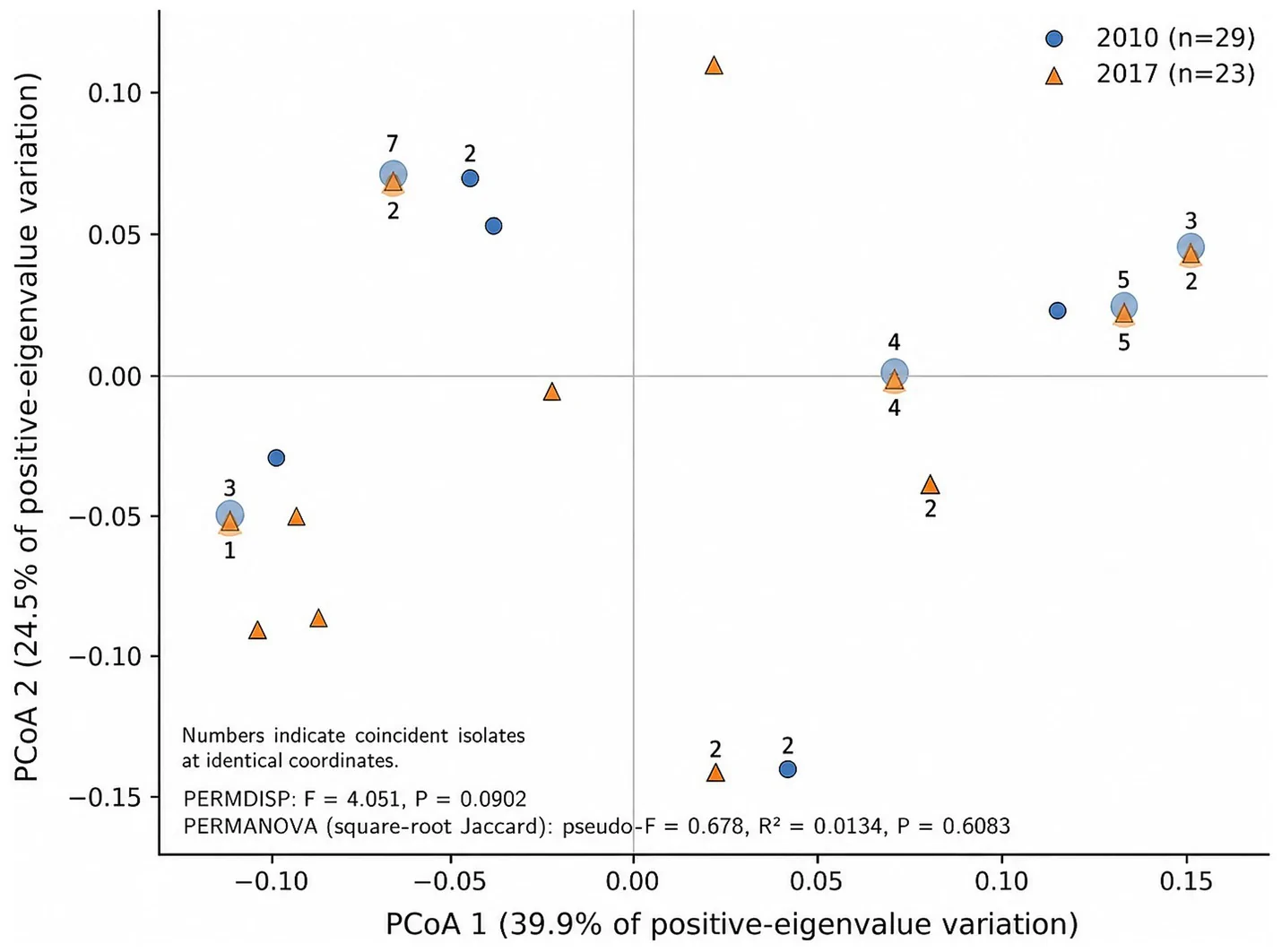
Principal coordinates analysis of square-root-transformed whole-virulome Jaccard dissimilarities among the 52 high-quality genomes. All 52 genomes are represented. Symbols indicate collection year. At coordinates representing multiple genomes, numeric labels adjacent to each symbol indicate the number of genomes from the corresponding collection year represented at that coordinate; a value of 1 is shown when a single genome from 1 year coincides with genomes from the other year. Unlabelled symbols represent single genomes at non-coincident coordinates. Point positions correspond to the original PCoA coordinates and were not jittered. The first two axes explain 39.9 and 24.5% of the positive-eigenvalue variation, respectively. No negative eigenvalues remained after square-root transformation. The same distance matrix was used for PERMDISP and PERMANOVA; PERMDISP did not reach statistical significance (*F* = 4.051, *p* = 0.0902), and the collection-year effect was not significant (PERMANOVA: Pseudo-*F* = 0.678, *R*^2^ = 0.0134, *p* = 0.6083; 9,999 permutations).

Total virulence-gene burden and module-completeness measures likewise showed no statistically significant collection-year differences after Benjamini-Hochberg correction (all *q* ≥ 0.724; Table 4). PCoA based on square-root-transformed Jaccard dissimilarities showed substantial intermingling of the 2010 and 2017 profiles, with the first two axes explaining 39.9 and 24.5% of the positive-eigenvalue variation, respectively (Figure 5). Multiple isolates shared identical or highly similar binary virulome profiles and consequently occupied identical or closely overlapping positions in the two-dimensional ordination. The untransformed Jaccard matrix generated 12 negative eigenvalues (most negative eigenvalue, −0.00641; summed absolute negative eigenvalues equivalent to 25.8% of the summed positive eigenvalues), whereas no negative eigenvalues remained after square-root transformation. PERMDISP did not reach statistical significance (*F* = 4.051, *p* = 0.0902). PERMANOVA using the same square-root-transformed Jaccard matrix showed that collection year explained only 1.34% of whole-virulome variation (pseudo-*F* = 0.678, *R*^2^ = 0.0134, *p* = 0.6083). No individual gene was associated with year after correction (minimum *q* = 1.000; Supplementary Table S4).

**Table 4:** Collection-year comparisons of total virulence-gene burden and module completeness.

Feature	2010 median (IQR)	2017 median (IQR)	*P*	q
Total virulence-gene count	228 (227.0–232.0)	232 (226.0–233.0)	0.595	0.968
Alginate and biofilm matrix	1 (1.000–1.000)	1 (1.000–1.000)	1.000	1.000
Type IV pili and adhesion	0.97 (0.939–0.970)	0.939 (0.939–0.970)	0.257	0.724
Flagellar motility	0.87 (0.870–1.000)	0.87 (0.870–1.000)	0.751	0.976
Lipopolysaccharide	0.714 (0.714–0.714)	0.714 (0.714–1.000)	0.073	0.724
Iron acquisition	0.848 (0.848–0.970)	0.848 (0.848–1.000)	0.471	0.968
Phenazine and pyocyanin	1 (1.000–1.000)	1 (1.000–1.000)	0.278	0.724
Quorum sensing and rhamnolipid	1 (1.000–1.000)	1 (1.000–1.000)	0.715	0.976
Type II secretion	1 (1.000–1.000)	1 (1.000–1.000)	1.000	1.000
Type III secretion apparatus	1 (1.000–1.000)	1 (1.000–1.000)	1.000	1.000
Type III secretion effectors	0.75 (0.750–0.750)	0.75 (0.750–0.750)	0.538	0.968
Type VI secretion	1 (1.000–1.000)	1 (1.000–1.000)	0.278	0.724
Secreted toxins and enzymes	1 (1.000–1.000)	1 (1.000–1.000)	0.115	0.724

Module completeness is the proportion of the cohort-defined union of genes in each functional module detected in an isolate. *p*-values are from two-sided Mann–Whitney *U* tests; *q*-values are Benjamini-Hochberg adjusted across the 13 aggregate comparisons.

### 3.8 Joint identity/coverage stringency affected selected gene calls

Most key findings were stable when the joint VFDB criterion was increased from 80% nucleotide identity and 80% reference coverage to 90% identity and 90% coverage. Universal or near-universal calls for *algD, alg8, alg44, pilT, fimV, pvdS, pchE, rhlA, rhlB, phzM, phzS, toxA, lasA, lasB, aprA, plcH, exoT, exoY, exoS, exoU, pscC, pscN, xcpQ, hcp1* and *icmF1/tssM1* were unchanged or changed minimally (Supplementary Figure S2; Supplementary Table S5). In contrast, *pilB* decreased from 52/52 to 13/52 and *pvdA* from 52/52 to 30/52 under the joint 90/90 criterion. The primary-threshold median identity was 88.5% for *pilB* but approximately 99.0% for *pvdA* (Table 2), suggesting that nucleotide identity plausibly contributed substantially to the reduction in *pilB* calls, whereas reference coverage may have contributed more strongly to the reduction in *pvdA* calls. Because identity and coverage were changed simultaneously, however, their individual contributions cannot be quantified from this sensitivity analysis. The results therefore demonstrate sensitivity of selected VFDB calls to overall homology-stringency rather than specifically to nucleotide-identity thresholds.

## 4 Discussion

This study defines the virulence-associated genomic architecture of an independent historical Hungarian collection of canine otitis-associated *P. aeruginosa*. The 52 high-quality genomes carried a dense, predominantly conserved virulence repertoire, whereas heterogeneity was concentrated in T3SS effectors and allele-diverse surface-structure and pyoverdine-associated loci. Neither total virulence-gene burden nor multivariate virulome composition differed detectably between 2010 and 2017. The conclusions regarding virulence-gene conservation, heterogeneity and collection-year comparisons are based on genomic prevalence and multivariate analyses. The previously generated core-genome topology is provided only as supplementary descriptive material and is not used as inferential evidence.

The external ear in chronic otitis is a selective environment characterized by epithelial disruption, altered lipid and moisture conditions, inflammatory exudate, repeated cleaning and frequent antimicrobial exposure. Persistence in this niche requires more than a single toxin. Conserved flagellar and type IV pilus systems support motility and initial attachment; alginate and other exopolysaccharide pathways support matrix production and spatial organization; siderophores secure iron under host limitation; quorum sensing coordinates density-dependent exoproducts; and secreted proteases, phospholipases and exotoxin A can damage tissue or modulate local defenses (9–15). The near-universal presence of these systems is therefore consistent with the broad ecological versatility of *P. aeruginosa* and with canine studies showing frequent strong biofilm formation (2, 7, 14, 15, 39).

The data should not be interpreted as proof that every gene was expressed in the diseased ear. VFDB screening identifies genomic homologues, not transcription, secretion, enzymatic activity or clinical severity. Biofilm formation, for example, depends on regulation, environmental conditions and lineage-specific matrix use; some strains rely more heavily on Pel than Psl, and gene presence alone does not predict the magnitude of a biofilm phenotype (14, 15). Nevertheless, conservation of the underlying machinery provides a credible genomic substrate for persistence. This distinction is clinically relevant: antimicrobial susceptibility testing addresses planktonic growth inhibition, whereas virulome content and biofilm physiology address different dimensions of pathogenesis and treatment failure (2, 40). Local control measures may therefore complement antimicrobial treatment. Stabilized hypochlorous acid has documented activity against *P. aeruginosa* and has been proposed for veterinary hygiene applications, although formulation-specific efficacy, biofilm activity, otic tolerability and clinical performance require direct validation (41). Antimicrobial peptides represent another potential non-conventional approach because of their membrane-targeting, immunomodulatory and anti-biofilm properties, but stability, delivery, toxicity and *in vivo* efficacy remain important translational limitations (42).

The T3SS apparatus was universal, but its effector repertoire was not. The dominant historical profile was *exoS*+/*exoU*−, consistent with the widespread distribution of *exoS*-positive lineages and with several canine datasets (7, 39). In the multinational study of 35 sequenced canine otitis isolates, *exoS* occurred in 24/35 and *exoU* in 5/35; one isolate carried both and five carried neither (7). Hattab et al. (39) detected *exoS* in 21/24 canine clinical isolates. Our cohort contained *exoS* calls in 49/52 and *exoU* calls in 4/52 genomes. One isolate showed assembly-level co-detection of both homologues. The assembled *exoU*-homologous sequence contained an apparent single-nucleotide insertion that would alter the predicted reading frame. However, because this observation derives from a long-read-only assembly and was not independently confirmed at read level or by Sanger sequencing, the apparent indel and associated frameshift cannot be distinguished confidently from residual sequencing or assembly error. This isolate should therefore be regarded as carrying genomic homologues of both *exoS* and *exoU*, without inference regarding the intactness or functional expression of the ExoU effector.

Comparison with the independent 2024 Hungarian cohort is informative but must remain descriptive because sampling, selection and quality-control structures differ. In the later collection, *exoS* was detected in 50/70 isolates and *exoU* in 15/70, compared with 49/52 and 4/52, respectively, in the present primary historical set (22). The proportion of *exoU*-positive isolates was descriptively higher in the 2024 cohort than in the historical cohort. However, because the cohorts differed in sampling, isolate selection and quality-control structure, this observation should not be interpreted as evidence of a temporal increase. The 2024 genomes were selected from a different clinical collection and no joint population model or harmonized sampling frame was available. A future analysis using all genomes under a single assembly, species-confirmation and variant-calling pipeline would be required to test temporal replacement formally.

The single genome showing *exoS/exoU* co-detection deserves cautious attention because these genes are commonly, but not absolutely, mutually exclusive (7, 21). Both homologues independently met the predefined VFDB calling criteria in a high-quality assembly and were therefore retained as a genomic observation. Targeted assembly-level inspection supported the presence of both homologous loci on separate contigs. However, no independent read-level, PCR or Sanger confirmation was available. In particular, the apparent single-base indel observed in the assembled *exoU*-homologous sequence remains unverified and may represent residual error in the long-read-only assembly. We therefore describe the finding strictly as genomic co-detection and make no inference regarding intact *exoU* coding sequence, functional co-expression or secretion competency (16, 21). The observation should therefore be regarded as an uncommon genomic configuration that warrants targeted validation in future studies.

Universal type II secretion components and near-universal extracellular-enzyme homologues indicate that genes associated with extracellular-protein secretion were broadly conserved across the collection. LasA, LasB, AprA, PlcH and exotoxin A act through distinct mechanisms, including proteolysis of host substrates, membrane damage and inhibition of protein synthesis (10–13). Their conservation may help explain why chronic pseudomonal otitis can remain destructive even when no single unusual toxin profile is present.

The HSI-I/HSI-1 type VI secretion backbone was also strongly conserved. T6SSs contribute primarily to interbacterial competition, niche occupancy and delivery of toxic effectors, although context-dependent interactions with eukaryotic hosts are also described (17, 43). In a polymicrobial or dysbiotic ear canal, such machinery may influence competition with resident bacteria and thereby support persistence. However, our VFDB-based module analysis did not comprehensively resolve the highly variable orphan effector and immunity islands described in human clinical populations (17). The present result should therefore be interpreted as conservation of the secretion scaffold, not identity of the full T6SS effector repertoire.

Several low-prevalence calls occurred within systems whose wider machinery was nearly complete. The major pilin reference *pilA* was detected in only two genomes, while *pilB*, *pilT*, *fimV* and other pilus components were widespread. Similarly, the reference *fliC* allele occurred in fewer than half of genomes despite universal or near-universal flagellar structural and regulatory genes. Pyoverdine-associated *pvdD*, *pvdE* and *fpvA* calls co-occurred in a subset, while the general pyoverdine regulatory and biosynthetic backbone remained conserved. These patterns are characteristic of allelic and structural diversity in surface-exposed or siderophore-recognition loci, where reference-specific nucleotide thresholds can separate homologous families (7, 10, 44).

The joint 90/90 sensitivity analysis showed that most major virulome conclusions were robust to increased homology-stringency, whereas selected loci, particularly *pilB* and *pvdA*, were sensitive to the stricter combined criterion. Importantly, because nucleotide identity and reference coverage were increased simultaneously, the observed reductions cannot be attributed uniquely to either parameter. The relatively low median identity of *pilB* at the primary threshold is consistent with an identity-driven effect, whereas the approximately 99% median identity of *pvdA* indicates that incomplete reference coverage is a plausible contributor to its reduced detection under the 90/90 criterion. Accordingly, these results are interpreted as evidence of threshold sensitivity of database-based gene calls rather than as evidence of biological gene loss or specifically of nucleotide-sequence divergence (25, 26).

The absence of a detectable difference in virulome composition between the 2010 and 2017 collections should not be interpreted as evidence that the underlying population was static. The study sampled only two discrete years, the isolates represented an archival convenience collection, and highly conserved binary virulome matrices have limited power to distinguish population-level temporal change. The appropriate conclusion is therefore narrower: no broad temporal restructuring of virulence-gene content was detected with the present dataset, sampling density and analytical framework. Although PERMDISP was not statistically significant (*p* = 0.0902), the unequal group sizes (29 versus 23 isolates) warrant cautious interpretation because some contribution of dispersion differences to the PERMANOVA result cannot be excluded.

From a One Health perspective, the most directly relevant context is provided by genomic studies of *Pseudomonas aeruginosa* from companion animals and humans. Recent canine WGS studies have identified internationally recognized high-risk sequence types also encountered in human nosocomial populations and have shown substantial genomic diversity among canine isolates, without evidence for a single host-restricted population structure (4, 7, 8). Additional cross-host evidence comes from genomic investigations documenting closely related *P. aeruginosa* isolates in companion animals, humans and shared environments, as well as recent companion-animal surveillance identifying internationally distributed high-risk clones with links to human-associated lineages (45, 46). These findings support the importance of host-resolved comparative genomics when evaluating the One Health relevance of veterinary *P. aeruginosa*. More broadly, studies of antimicrobial resistance in wild birds, poultry and other farm animals illustrate how antimicrobial-resistance determinants and mobile genetic elements can occur across diverse animal reservoirs and reinforce the wider value of integrated One Health surveillance (47–49). Long-term antimicrobial-resistance trends in canine otitis pathogens further emphasize the clinical relevance of such surveillance in companion animals (50), while broader genomic frameworks provide a methodological basis for investigating antimicrobial resistance across One Health interfaces (51). The present study did not include human or environmental comparator genomes and therefore cannot establish cross-host transmission or zoonotic directionality. Its contribution is restricted to documenting virulence-associated genomic diversity within a veterinary clinical population; any assessment of cross-host risk should integrate comparative phylogenomics, sequence type, antimicrobial resistance, mobile genetic elements and epidemiological exposure data rather than virulence-gene presence alone.

The principal strengths are the use of an independent historical collection, long-read assemblies, explicit genome-quality filtering, a prespecified identity/coverage threshold, isolate-level source tables, false-discovery-rate control and sensitivity analysis at a stricter VFDB threshold. The distinction between a 52-genome primary set and seven QC-flagged genomes prevented incomplete assemblies from inflating absence estimates. Exclusion of a species-discordant assembly further protected the species-specific prevalence analysis from a major taxonomic outlier.

Several limitations remain. The collection was retrospective and not population based and therefore does not estimate national prevalence. Although only one isolate per dog was included, clinic-level identifiers and sufficiently detailed geographical metadata were unavailable, and the equivalence of the underlying source populations sampled in 2010 and 2017 cannot be demonstrated retrospectively. In addition, genomic data were unavailable for 10 of the 70 nominal isolate identifiers, and the historical records did not permit the reasons for this missingness to be reconstructed. Because the possibility of non-random assembly availability cannot be excluded, some selection bias in the genome-based analytical cohort is therefore possible. Consequently, collection-year comparisons may be influenced by unmeasured differences in the populations submitting diagnostic samples and should not be interpreted as population-level temporal surveillance. Clinical metadata were also insufficient to associate virulome patterns with disease severity, chronicity, prior treatment, laterality, biofilm phenotype or outcome. Only two discrete sampling years were represented, limiting statistical power for temporal inference and precluding continuous trend modelling. Long-read-only assemblies may retain residual base errors after polishing, and no independent short-read confirmation or targeted PCR validation was available. Genome-based species confirmation was applied to all 59 retained assemblies, each of which exceeded the conventional 95% ANI boundary to PAO1. Isolate 134 was the sole taxonomically discordant assembly and was excluded before all *P. aeruginosa*-specific analyses. Its alternative species identity was not determined and is not inferred in the present study. VFDB calls are reference dependent and cannot distinguish pseudogenes, regulatory disruption, expression or secretion. Module completeness was defined against the cohort union rather than a curated essential-gene set. Because the core-genome tree was inferred without bootstrap or SH-aLRT resampling and was pruned from a wider analysis, it was restricted to descriptive visualization and was not used for formal clade, lineage, population-structure or transmission inference. Dedicated branch-supported phylogenomics based on a harmonized cohort would be required for such questions. Finally, the comparison with the 2024 Hungarian cohort was descriptive because the cohorts were not assembled through a unified sampling and analytical design.

## 5 Conclusion

Historical canine otitis-associated *P. aeruginosa* isolates from Hungary carried a dense and highly conserved virulence-associated genomic backbone encompassing loci associated with biofilm matrix formation, adhesion and motility, iron acquisition, quorum sensing, phenazine and rhamnolipid biosynthesis, extracellular enzymes, and type II, type III and type VI secretion systems. Strain-level heterogeneity was concentrated in T3SS effectors and allele-diverse surface or siderophore loci. The dominant profile was *exoS*+/*exoU*-, while *exoU* occurred in a small subset and one high-quality genome showed genomic co-detection of *exoS* and an *exoU* homologue. No statistically detectable difference in virulome composition was observed between the 2010 and 2017 collections. Overall, the data demonstrate a broadly conserved virulence-associated genomic repertoire accompanied by isolate-level heterogeneity in T3SS effector homologues and selected surface- and siderophore-associated loci. These genomic detections should not be interpreted as direct evidence of gene expression, protein production or functional pathogenicity. Functional studies integrating gene expression, secretion phenotypes, biofilm characteristics and clinical metadata will be required to determine how the identified genomic variation contributes to persistence and disease phenotype.

## Data Availability

The datasets presented in this study can be found in online repositories. The names of the repository/repositories and accession number(s) can be found in the article/Supplementary material.
