# Supplementary material for: A conserved virulence backbone and heterogeneous type III secretion effector profiles in historical canine otitis-associated Pseudomonas aeruginosa from Hungary
Source: Front Vet Sci. 2026 Sep 10;13:1951261. doi: 10.3389/fvets.2026.1951261 (PMC13601786; doi:10.3389/fvets.2026.1951261)
Supplement: Supplementary file 1 [file Supplementary_file_1.docx]

Supplementary Material

Supplementary Table S2. Prevalence, median nucleotide identity and median reference coverage of all 242 VFDB-associated genes detected in the 52 high-quality genomes.

| **Broad module** | **Gene** | **VFDB factor** | **n** | **%** | **Identity** | **Coverage** |
| --- | --- | --- | --- | --- | --- | --- |
| Alginate and biofilm matrix | *alg44* | Alginate (VF0091) | 52 | 100.0 | 99.7 | 100.0 |
| Alginate and biofilm matrix | *alg8* | Alginate (VF0091) | 52 | 100.0 | 99.9 | 100.0 |
| Alginate and biofilm matrix | *algA* | Alginate (VF0091) | 52 | 100.0 | 99.8 | 100.0 |
| Alginate and biofilm matrix | *algB* | Alginate (VF0091) | 52 | 100.0 | 99.9 | 100.0 |
| Alginate and biofilm matrix | *algC* | Alginate biosynthesis (CVF522) | 52 | 100.0 | 99.9 | 100.0 |
| Alginate and biofilm matrix | *algD* | Alginate (VF0091) | 52 | 100.0 | 100.0 | 100.0 |
| Alginate and biofilm matrix | *algE* | Alginate (VF0091) | 52 | 100.0 | 99.6 | 100.0 |
| Alginate and biofilm matrix | *algF* | Alginate (VF0091) | 52 | 100.0 | 99.7 | 100.0 |
| Alginate and biofilm matrix | *algG* | Alginate (VF0091) | 52 | 100.0 | 99.9 | 100.0 |
| Alginate and biofilm matrix | *algI* | Alginate (VF0091) | 52 | 100.0 | 99.7 | 100.0 |
| Alginate and biofilm matrix | *algJ* | Alginate (VF0091) | 52 | 100.0 | 99.8 | 100.0 |
| Alginate and biofilm matrix | *algK* | Alginate (VF0091) | 52 | 100.0 | 99.8 | 100.0 |
| Alginate and biofilm matrix | *algL* | Alginate (VF0091) | 52 | 100.0 | 99.8 | 100.0 |
| Alginate and biofilm matrix | *algP/algR3* | Alginate (VF0091) | 52 | 100.0 | 99.1 | 100.0 |
| Alginate and biofilm matrix | *algQ* | Alginate (VF0091) | 52 | 100.0 | 99.6 | 100.0 |
| Alginate and biofilm matrix | *algR* | Alginate (VF0091) | 52 | 100.0 | 99.7 | 100.0 |
| Alginate and biofilm matrix | *algU* | Alginate (VF0091) | 52 | 100.0 | 99.8 | 100.0 |
| Alginate and biofilm matrix | *algW* | Alginate regulation (CVF523) | 52 | 100.0 | 99.7 | 100.0 |
| Alginate and biofilm matrix | *algX* | Alginate (VF0091) | 52 | 100.0 | 99.7 | 100.0 |
| Alginate and biofilm matrix | *algZ* | Alginate (VF0091) | 52 | 100.0 | 99.8 | 100.0 |
| Alginate and biofilm matrix | *mucA* | Alginate (VF0091) | 52 | 100.0 | 99.8 | 100.0 |
| Alginate and biofilm matrix | *mucB* | Alginate (VF0091) | 52 | 100.0 | 99.9 | 100.0 |
| Alginate and biofilm matrix | *mucC* | Alginate (VF0091) | 52 | 100.0 | 99.8 | 100.0 |
| Alginate and biofilm matrix | *mucD* | Alginate regulation (CVF523) | 52 | 100.0 | 99.6 | 100.0 |
| Alginate and biofilm matrix | *mucE* | Alginate regulation (CVF523) | 52 | 100.0 | 99.6 | 100.0 |
| Alginate and biofilm matrix | *mucP* | Alginate regulation (CVF523) | 52 | 100.0 | 99.7 | 100.0 |
| Flagellar motility | *fleN* | Flagella (VF0273) | 52 | 100.0 | 99.9 | 100.0 |
| Flagellar motility | *fleQ* | Flagella (VF0273) | 52 | 100.0 | 99.2 | 100.0 |
| Flagellar motility | *fleR* | Flagella (VF0273) | 52 | 100.0 | 99.4 | 100.0 |
| Flagellar motility | *fleS* | Deoxyhexose linking sugar 209 Da capping structure (AI138) | 52 | 100.0 | 99.3 | 100.0 |
| Flagellar motility | *flgA* | Deoxyhexose linking sugar 209 Da capping structure (AI138) | 52 | 100.0 | 99.9 | 100.0 |
| Flagellar motility | *flgB* | Deoxyhexose linking sugar 209 Da capping structure (AI138) | 52 | 100.0 | 100.0 | 100.0 |
| Flagellar motility | *flgC* | Flagella (VF0273) | 52 | 100.0 | 100.0 | 100.0 |
| Flagellar motility | *flgD* | Flagella (VF0273) | 52 | 100.0 | 99.9 | 100.0 |
| Flagellar motility | *flgE* | Flagella (VF0273) | 52 | 100.0 | 99.6 | 100.0 |
| Flagellar motility | *flgF* | Flagella (VF0273) | 52 | 100.0 | 100.0 | 100.0 |
| Flagellar motility | *flgG* | Flagella (VF0273) | 52 | 100.0 | 99.8 | 100.0 |
| Flagellar motility | *flgH* | Flagella (VF0273) | 52 | 100.0 | 99.6 | 100.0 |
| Flagellar motility | *flgI* | Flagella (VF0273) | 52 | 100.0 | 99.6 | 100.0 |
| Flagellar motility | *flgJ* | Flagella (VF0273) | 52 | 100.0 | 99.7 | 100.0 |
| Flagellar motility | *flgK* | Flagella (VF0273) | 52 | 100.0 | 86.7 | 99.7 |
| Flagellar motility | *flgM* | Deoxyhexose linking sugar 209 Da capping structure (AI138) | 52 | 100.0 | 99.4 | 100.0 |
| Flagellar motility | *flgN* | Deoxyhexose linking sugar 209 Da capping structure (AI138) | 52 | 100.0 | 99.2 | 100.0 |
| Flagellar motility | *flhA* | Flagella (VF0273) | 52 | 100.0 | 99.7 | 100.0 |
| Flagellar motility | *flhB* | Flagella (VF0273) | 52 | 100.0 | 99.7 | 100.0 |
| Flagellar motility | *flhF* | Flagella (VF0273) | 52 | 100.0 | 99.6 | 100.0 |
| Flagellar motility | *fliA* | Deoxyhexose linking sugar 209 Da capping structure (AI138) | 52 | 100.0 | 99.9 | 100.0 |
| Flagellar motility | *fliE* | Flagella (VF0273) | 52 | 100.0 | 99.4 | 100.0 |
| Flagellar motility | *fliF* | Flagella (VF0273) | 52 | 100.0 | 99.7 | 100.0 |
| Flagellar motility | *fliG* | Flagella (VF0273) | 52 | 100.0 | 99.8 | 100.0 |
| Flagellar motility | *fliH* | Flagella (VF0273) | 52 | 100.0 | 99.6 | 100.0 |
| Flagellar motility | *fliI* | Flagella (VF0273) | 52 | 100.0 | 99.3 | 100.0 |
| Flagellar motility | *fliJ* | Flagella (VF0273) | 52 | 100.0 | 99.8 | 100.0 |
| Flagellar motility | *fliK* | Deoxyhexose linking sugar 209 Da capping structure (AI138) | 52 | 100.0 | 98.9 | 100.0 |
| Flagellar motility | *fliL* | Deoxyhexose linking sugar 209 Da capping structure (AI138) | 52 | 100.0 | 100.0 | 100.0 |
| Flagellar motility | *fliM* | Flagella (VF0273) | 52 | 100.0 | 99.9 | 100.0 |
| Flagellar motility | *fliN* | Flagella (VF0273) | 52 | 100.0 | 99.8 | 100.0 |
| Flagellar motility | *fliO* | Flagella (VF0273) | 52 | 100.0 | 99.8 | 100.0 |
| Flagellar motility | *fliP* | Flagella (VF0273) | 52 | 100.0 | 99.9 | 100.0 |
| Flagellar motility | *fliQ* | Flagella (VF0273) | 52 | 100.0 | 99.6 | 100.0 |
| Flagellar motility | *fliR* | Flagella (VF0273) | 52 | 100.0 | 99.5 | 100.0 |
| Flagellar motility | *motA* | Deoxyhexose linking sugar 209 Da capping structure (AI138) | 52 | 100.0 | 99.8 | 100.0 |
| Flagellar motility | *motB* | Deoxyhexose linking sugar 209 Da capping structure (AI138) | 52 | 100.0 | 99.4 | 100.0 |
| Flagellar motility | *motC* | Deoxyhexose linking sugar 209 Da capping structure (AI138) | 52 | 100.0 | 99.9 | 100.0 |
| Flagellar motility | *motD* | Deoxyhexose linking sugar 209 Da capping structure (AI138) | 52 | 100.0 | 99.8 | 100.0 |
| Flagellar motility | *motY* | Deoxyhexose linking sugar 209 Da capping structure (AI138) | 52 | 100.0 | 99.6 | 100.0 |
| Flagellar motility | *fliC* | Flagella (VF0273) | 24 | 46.2 | 99.9 | 100.0 |
| Flagellar motility | *fleI/flag* | Deoxyhexose linking sugar 209 Da capping structure (AI138) | 23 | 44.2 | 99.7 | 100.0 |
| Flagellar motility | *fleP* | Deoxyhexose linking sugar 209 Da capping structure (AI138) | 23 | 44.2 | 100.0 | 100.0 |
| Flagellar motility | *flgL* | Flagella (VF0273) | 23 | 44.2 | 99.8 | 100.0 |
| Flagellar motility | *fliD* | Flagella (VF0273) | 23 | 44.2 | 99.9 | 100.0 |
| Flagellar motility | *fliS* | Deoxyhexose linking sugar 209 Da capping structure (AI138) | 23 | 44.2 | 99.7 | 100.0 |
| Iron acquisition | *fptA* | Pyochelin (VF0095) | 52 | 100.0 | 99.4 | 100.0 |
| Iron acquisition | *mbtH-like* | pyoverdine (IA001) | 52 | 100.0 | 100.0 | 100.0 |
| Iron acquisition | *pchA* | Pyochelin (VF0095) | 52 | 100.0 | 99.4 | 100.0 |
| Iron acquisition | *pchB* | Pyochelin (VF0095) | 52 | 100.0 | 99.7 | 100.0 |
| Iron acquisition | *pchC* | Pyochelin (VF0095) | 52 | 100.0 | 99.7 | 100.0 |
| Iron acquisition | *pchD* | Pyochelin (VF0095) | 52 | 100.0 | 99.5 | 100.0 |
| Iron acquisition | *pchF* | Pyochelin (VF0095) | 52 | 100.0 | 99.4 | 100.0 |
| Iron acquisition | *pchG* | Pyochelin (VF0095) | 52 | 100.0 | 99.3 | 100.0 |
| Iron acquisition | *pchH* | Pyochelin (VF0095) | 52 | 100.0 | 99.2 | 100.0 |
| Iron acquisition | *pchI* | Pyochelin (VF0095) | 52 | 100.0 | 99.0 | 100.0 |
| Iron acquisition | *pchR* | Pyochelin (VF0095) | 52 | 100.0 | 99.7 | 100.0 |
| Iron acquisition | *ptxR* | pyoverdine (IA001) | 52 | 100.0 | 99.7 | 100.0 |
| Iron acquisition | *pvcA* | pyoverdine (IA001) | 52 | 100.0 | 99.6 | 100.0 |
| Iron acquisition | *pvcB* | pyoverdine (IA001) | 52 | 100.0 | 99.8 | 100.0 |
| Iron acquisition | *pvcC* | pyoverdine (IA001) | 52 | 100.0 | 99.5 | 100.0 |
| Iron acquisition | *pvcD* | pyoverdine (IA001) | 52 | 100.0 | 99.8 | 100.0 |
| Iron acquisition | *pvdA* | Pyoverdine (VF0094) | 52 | 100.0 | 99.0 | 100.0 |
| Iron acquisition | *pvdF* | pyoverdine (IA001) | 52 | 100.0 | 90.3 | 100.0 |
| Iron acquisition | *pvdG* | pyoverdine (IA001) | 52 | 100.0 | 99.2 | 100.0 |
| Iron acquisition | *pvdH* | pyoverdine (IA001) | 52 | 100.0 | 99.5 | 100.0 |
| Iron acquisition | *pvdL* | pyoverdine (IA001) | 52 | 100.0 | 99.3 | 100.0 |
| Iron acquisition | *pvdM* | pyoverdine (IA001) | 52 | 100.0 | 94.2 | 99.8 |
| Iron acquisition | *pvdN* | pyoverdine (IA001) | 52 | 100.0 | 99.4 | 100.0 |
| Iron acquisition | *pvdO* | pyoverdine (IA001) | 52 | 100.0 | 97.8 | 100.0 |
| Iron acquisition | *pvdP* | pyoverdine (IA001) | 52 | 100.0 | 84.9 | 100.0 |
| Iron acquisition | *pvdQ* | pyoverdine (IA001) | 52 | 100.0 | 99.5 | 100.0 |
| Iron acquisition | *pvdS* | Pyoverdine (VF0094) | 52 | 100.0 | 99.5 | 100.0 |
| Iron acquisition | *pchE* | Pyochelin (VF0095) | 51 | 98.1 | 99.5 | 100.0 |
| Iron acquisition | *fpvA* | Pyoverdine (VF0094) | 16 | 30.8 | 99.8 | 100.0 |
| Iron acquisition | *pvdD* | Pyoverdine (VF0094) | 16 | 30.8 | 99.5 | 100.0 |
| Iron acquisition | *pvdE* | Pyoverdine (VF0094) | 16 | 30.8 | 99.7 | 100.0 |
| Iron acquisition | *pvdI* | pyoverdine (IA001) | 16 | 30.8 | 99.0 | 100.0 |
| Iron acquisition | *pvdJ* | pyoverdine (IA001) | 15 | 28.8 | 99.5 | 100.0 |
| Lipopolysaccharide | *waaA* | LPS (VF0085) | 52 | 100.0 | 99.8 | 100.0 |
| Lipopolysaccharide | *waaC* | LPS (VF0085) | 52 | 100.0 | 99.5 | 100.0 |
| Lipopolysaccharide | *waaF* | LPS (VF0085) | 52 | 100.0 | 99.8 | 100.0 |
| Lipopolysaccharide | *waaG* | LPS (VF0085) | 52 | 100.0 | 99.5 | 100.0 |
| Lipopolysaccharide | *waaP* | LPS (VF0085) | 52 | 100.0 | 99.6 | 100.0 |
| Lipopolysaccharide | *wzy* | LPS (VF0085) | 10 | 19.2 | 100.0 | 100.0 |
| Lipopolysaccharide | *wzz* | LPS (VF0085) | 10 | 19.2 | 100.0 | 100.0 |
| Phenazine and pyocyanin | *phzA1* | Phenazines biosynthesis (CVF536) | 52 | 100.0 | 99.8 | 100.0 |
| Phenazine and pyocyanin | *phzB1* | Phenazines biosynthesis (CVF536) | 52 | 100.0 | 98.8 | 100.0 |
| Phenazine and pyocyanin | *phzC1* | Phenazines biosynthesis (CVF536) | 52 | 100.0 | 99.5 | 100.0 |
| Phenazine and pyocyanin | *phzD1* | Phenazines biosynthesis (CVF536) | 52 | 100.0 | 99.3 | 100.0 |
| Phenazine and pyocyanin | *phzE1* | Phenazines biosynthesis (CVF536) | 52 | 100.0 | 99.6 | 100.0 |
| Phenazine and pyocyanin | *phzF1* | Phenazines biosynthesis (CVF536) | 52 | 100.0 | 99.5 | 100.0 |
| Phenazine and pyocyanin | *phzG1* | Phenazines biosynthesis (CVF536) | 52 | 100.0 | 99.8 | 100.0 |
| Phenazine and pyocyanin | *phzM* | Pyocyanin (VF0100) | 52 | 100.0 | 99.7 | 100.0 |
| Phenazine and pyocyanin | *phzS* | Pyocyanin (VF0100) | 52 | 100.0 | 99.3 | 100.0 |
| Phenazine and pyocyanin | *phzH* | Phenazines biosynthesis (CVF536) | 51 | 98.1 | 99.6 | 100.0 |
| Quorum sensing and rhamnolipid | *rhlA* | Rhamnolipid (VF0089) | 52 | 100.0 | 99.5 | 100.0 |
| Quorum sensing and rhamnolipid | *rhlB* | Rhamnolipid (VF0089) | 52 | 100.0 | 99.4 | 100.0 |
| Quorum sensing and rhamnolipid | *rhlC* | Rhamnolipid biosynthesis (CVF524) | 52 | 100.0 | 99.4 | 100.0 |
| Quorum sensing and rhamnolipid | *rhlI* | Quorum sensing (VF0093) | 51 | 98.1 | 98.5 | 100.0 |
| Quorum sensing and rhamnolipid | *lasI* | Quorum sensing (VF0093) | 50 | 96.2 | 100.0 | 100.0 |
| Secreted toxins and enzymes | *aprA* | Alkaline protease (VF0090) | 52 | 100.0 | 99.6 | 100.0 |
| Secreted toxins and enzymes | *lasA* | LasA (VF0088) | 52 | 100.0 | 99.3 | 100.0 |
| Secreted toxins and enzymes | *lasB* | LasB (VF0087) | 52 | 100.0 | 99.7 | 100.0 |
| Secreted toxins and enzymes | *plcH* | PLC (VF0092) | 51 | 98.1 | 99.7 | 100.0 |
| Secreted toxins and enzymes | *toxA* | ExoA (VF0086) | 51 | 98.1 | 99.3 | 100.0 |
| Type II secretion | *xcpP* | xcp secretion system (VF0084) | 52 | 100.0 | 100.0 | 100.0 |
| Type II secretion | *xcpQ* | xcp secretion system (VF0084) | 52 | 100.0 | 99.6 | 100.0 |
| Type II secretion | *xcpR* | xcp secretion system (VF0084) | 52 | 100.0 | 99.5 | 100.0 |
| Type II secretion | *xcpS* | xcp secretion system (VF0084) | 52 | 100.0 | 99.6 | 100.0 |
| Type II secretion | *xcpT* | xcp secretion system (VF0084) | 52 | 100.0 | 99.5 | 100.0 |
| Type II secretion | *xcpU* | xcp secretion system (VF0084) | 52 | 100.0 | 99.8 | 100.0 |
| Type II secretion | *xcpV* | xcp secretion system (VF0084) | 52 | 100.0 | 99.5 | 100.0 |
| Type II secretion | *xcpW* | xcp secretion system (VF0084) | 52 | 100.0 | 99.6 | 100.0 |
| Type II secretion | *xcpX* | xcp secretion system (VF0084) | 52 | 100.0 | 99.6 | 100.0 |
| Type II secretion | *xcpY* | xcp secretion system (VF0084) | 52 | 100.0 | 99.7 | 100.0 |
| Type II secretion | *xcpZ* | xcp secretion system (VF0084) | 52 | 100.0 | 99.2 | 100.0 |
| Type III secretion apparatus | *exsA* | TTSS (VF0083) | 52 | 100.0 | 99.9 | 100.0 |
| Type III secretion apparatus | *exsB* | TTSS (VF0083) | 52 | 100.0 | 99.4 | 100.0 |
| Type III secretion apparatus | *exsC* | TTSS (VF0083) | 52 | 100.0 | 99.1 | 100.0 |
| Type III secretion apparatus | *exsD* | TTSS (VF0083) | 52 | 100.0 | 99.6 | 100.0 |
| Type III secretion apparatus | *exsE* | TTSS (VF0083) | 52 | 100.0 | 99.2 | 100.0 |
| Type III secretion apparatus | *pcr1* | TTSS (VF0083) | 52 | 100.0 | 100.0 | 100.0 |
| Type III secretion apparatus | *pcr2* | TTSS (VF0083) | 52 | 100.0 | 98.9 | 100.0 |
| Type III secretion apparatus | *pcr3* | TTSS (VF0083) | 52 | 100.0 | 99.7 | 100.0 |
| Type III secretion apparatus | *pcr4* | TTSS (VF0083) | 52 | 100.0 | 99.7 | 100.0 |
| Type III secretion apparatus | *pcrD* | TTSS (VF0083) | 52 | 100.0 | 99.6 | 100.0 |
| Type III secretion apparatus | *pcrG* | TTSS (VF0083) | 52 | 100.0 | 99.7 | 100.0 |
| Type III secretion apparatus | *pcrH* | TTSS (VF0083) | 52 | 100.0 | 99.2 | 100.0 |
| Type III secretion apparatus | *pcrR* | TTSS (VF0083) | 52 | 100.0 | 100.0 | 100.0 |
| Type III secretion apparatus | *pcrV* | TTSS (VF0083) | 52 | 100.0 | 99.7 | 100.0 |
| Type III secretion apparatus | *popB* | TTSS (VF0083) | 52 | 100.0 | 99.6 | 100.0 |
| Type III secretion apparatus | *popD* | TTSS (VF0083) | 52 | 100.0 | 99.8 | 100.0 |
| Type III secretion apparatus | *popN* | TTSS (VF0083) | 52 | 100.0 | 99.3 | 100.0 |
| Type III secretion apparatus | *pscB* | TTSS (VF0083) | 52 | 100.0 | 99.5 | 100.0 |
| Type III secretion apparatus | *pscC* | TTSS (VF0083) | 52 | 100.0 | 99.7 | 100.0 |
| Type III secretion apparatus | *pscD* | TTSS (VF0083) | 52 | 100.0 | 99.7 | 100.0 |
| Type III secretion apparatus | *pscE* | TTSS (VF0083) | 52 | 100.0 | 99.0 | 100.0 |
| Type III secretion apparatus | *pscF* | TTSS (VF0083) | 52 | 100.0 | 100.0 | 100.0 |
| Type III secretion apparatus | *pscG* | TTSS (VF0083) | 52 | 100.0 | 100.0 | 100.0 |
| Type III secretion apparatus | *pscH* | TTSS (VF0083) | 52 | 100.0 | 99.8 | 100.0 |
| Type III secretion apparatus | *pscI* | TTSS (VF0083) | 52 | 100.0 | 99.7 | 100.0 |
| Type III secretion apparatus | *pscJ* | TTSS (VF0083) | 52 | 100.0 | 99.6 | 100.0 |
| Type III secretion apparatus | *pscK* | TTSS (VF0083) | 52 | 100.0 | 98.6 | 99.0 |
| Type III secretion apparatus | *pscL* | TTSS (VF0083) | 52 | 100.0 | 99.8 | 100.0 |
| Type III secretion apparatus | *pscN* | TTSS (VF0083) | 52 | 100.0 | 99.0 | 100.0 |
| Type III secretion apparatus | *pscO* | TTSS (VF0083) | 52 | 100.0 | 99.4 | 100.0 |
| Type III secretion apparatus | *pscP* | TTSS (VF0083) | 52 | 100.0 | 97.4 | 99.5 |
| Type III secretion apparatus | *pscQ* | TTSS (VF0083) | 52 | 100.0 | 99.2 | 100.0 |
| Type III secretion apparatus | *pscR* | TTSS (VF0083) | 52 | 100.0 | 99.6 | 100.0 |
| Type III secretion apparatus | *pscS* | TTSS (VF0083) | 52 | 100.0 | 99.6 | 100.0 |
| Type III secretion apparatus | *pscT* | TTSS (VF0083) | 52 | 100.0 | 99.2 | 100.0 |
| Type III secretion apparatus | *pscU* | TTSS (VF0083) | 52 | 100.0 | 99.5 | 100.0 |
| Type III secretion effectors | *exoT* | ExoT (VF0097) | 52 | 100.0 | 99.1 | 100.0 |
| Type III secretion effectors | *exoY* | ExoY (VF0099) | 50 | 96.2 | 99.6 | 100.0 |
| Type III secretion effectors | *exoS* | ExoS (VF0096) | 49 | 94.2 | 99.6 | 100.0 |
| Type III secretion effectors | *exoU* | ExoU (VF0098) | 4 | 7.7 | 99.9 | 100.0 |
| Type IV pili and adhesion | *chpA* | Type IV pili (VF0082) | 52 | 100.0 | 99.6 | 100.0 |
| Type IV pili and adhesion | *chpB* | Type IV pili (VF0082) | 52 | 100.0 | 99.1 | 100.0 |
| Type IV pili and adhesion | *chpC* | Type IV pili (VF0082) | 52 | 100.0 | 98.8 | 100.0 |
| Type IV pili and adhesion | *chpD* | Type IV pili (VF0082) | 52 | 100.0 | 99.4 | 100.0 |
| Type IV pili and adhesion | *chpE* | Type IV pili (VF0082) | 52 | 100.0 | 99.3 | 100.0 |
| Type IV pili and adhesion | *fimV* | Type IV pili (VF0082) | 52 | 100.0 | 99.3 | 100.0 |
| Type IV pili and adhesion | *pilB* | Type IV pili (VF0082) | 52 | 100.0 | 88.5 | 100.0 |
| Type IV pili and adhesion | *pilF* | Type IV pili (VF0082) | 52 | 100.0 | 99.7 | 100.0 |
| Type IV pili and adhesion | *pilG* | Type IV pili (VF0082) | 52 | 100.0 | 100.0 | 100.0 |
| Type IV pili and adhesion | *pilH* | Type IV pili (VF0082) | 52 | 100.0 | 100.0 | 100.0 |
| Type IV pili and adhesion | *pilI* | Type IV pili (VF0082) | 52 | 100.0 | 100.0 | 100.0 |
| Type IV pili and adhesion | *pilJ* | Type IV pili (VF0082) | 52 | 100.0 | 99.7 | 100.0 |
| Type IV pili and adhesion | *pilK* | Type IV pili (VF0082) | 52 | 100.0 | 99.5 | 100.0 |
| Type IV pili and adhesion | *pilM* | Type IV pili (VF0082) | 52 | 100.0 | 99.8 | 100.0 |
| Type IV pili and adhesion | *pilN* | Type IV pili (VF0082) | 52 | 100.0 | 99.8 | 100.0 |
| Type IV pili and adhesion | *pilO* | Type IV pili (VF0082) | 52 | 100.0 | 99.8 | 100.0 |
| Type IV pili and adhesion | *pilP* | Type IV pili (VF0082) | 52 | 100.0 | 100.0 | 100.0 |
| Type IV pili and adhesion | *pilR* | Type IV pili (VF0082) | 52 | 100.0 | 99.8 | 100.0 |
| Type IV pili and adhesion | *pilS* | Type IV pili (VF0082) | 52 | 100.0 | 99.6 | 100.0 |
| Type IV pili and adhesion | *pilT* | Type IV pili (VF0082) | 52 | 100.0 | 99.7 | 100.0 |
| Type IV pili and adhesion | *pilU* | Type IV pili (VF0082) | 52 | 100.0 | 99.9 | 100.0 |
| Type IV pili and adhesion | *xcpA/pilD* | Type IV pili (VF0082) | 52 | 100.0 | 99.4 | 100.0 |
| Type IV pili and adhesion | *pilQ* | Type IV pili (VF0082) | 51 | 98.1 | 97.1 | 100.0 |
| Type IV pili and adhesion | *pilE* | Type IV pili (VF0082) | 45 | 86.5 | 99.5 | 100.0 |
| Type IV pili and adhesion | *pilX* | Type IV pili (VF0082) | 45 | 86.5 | 99.8 | 100.0 |
| Type IV pili and adhesion | *pilY1* | Type IV pili (VF0082) | 45 | 86.5 | 92.2 | 99.7 |
| Type IV pili and adhesion | *pilY2* | Type IV pili (VF0082) | 45 | 86.5 | 100.0 | 100.0 |
| Type IV pili and adhesion | *fimT* | Type IV pili (VF0082) | 44 | 84.6 | 99.4 | 100.0 |
| Type IV pili and adhesion | *fimU* | Type IV pili (VF0082) | 44 | 84.6 | 99.8 | 100.0 |
| Type IV pili and adhesion | *pilV* | Type IV pili (VF0082) | 44 | 84.6 | 99.8 | 100.0 |
| Type IV pili and adhesion | *pilW* | Type IV pili (VF0082) | 44 | 84.6 | 99.8 | 100.0 |
| Type IV pili and adhesion | *pilC* | Type IV pili (VF0082) | 23 | 44.2 | 80.0 | 98.7 |
| Type IV pili and adhesion | *pilA* | Type IV pili (VF0082) | 2 | 3.8 | 86.6 | 99.2 |
| Type VI secretion | *clpV1* | HSI-I (VF0334) | 52 | 100.0 | 99.6 | 100.0 |
| Type VI secretion | *dotU1* | HSI-I (VF0334) | 52 | 100.0 | 99.8 | 100.0 |
| Type VI secretion | *fha1* | HSI-I (VF0334) | 52 | 100.0 | 99.0 | 100.0 |
| Type VI secretion | *hcp1* | HSI-I (VF0334) | 52 | 100.0 | 100.0 | 100.0 |
| Type VI secretion | *hsiA1* | HSI-I (VF0334) | 52 | 100.0 | 98.8 | 100.0 |
| Type VI secretion | *hsiB1/vipA* | HSI-I (VF0334) | 52 | 100.0 | 99.8 | 100.0 |
| Type VI secretion | *hsiC1/vipB* | HSI-I (VF0334) | 52 | 100.0 | 99.7 | 100.0 |
| Type VI secretion | *hsiE1* | HSI-I (VF0334) | 52 | 100.0 | 99.5 | 100.0 |
| Type VI secretion | *hsiF1* | HSI-I (VF0334) | 52 | 100.0 | 99.8 | 100.0 |
| Type VI secretion | *hsiG1* | HSI-I (VF0334) | 52 | 100.0 | 99.8 | 100.0 |
| Type VI secretion | *hsiH1* | HSI-I (VF0334) | 52 | 100.0 | 99.9 | 100.0 |
| Type VI secretion | *hsiJ1* | HSI-I (VF0334) | 52 | 100.0 | 99.5 | 100.0 |
| Type VI secretion | *icmF1/tssM1* | HSI-I (VF0334) | 52 | 100.0 | 99.5 | 100.0 |
| Type VI secretion | *lip1* | HSI-I (VF0334) | 52 | 100.0 | 99.8 | 100.0 |
| Type VI secretion | *ppkA* | HSI-I (VF0334) | 52 | 100.0 | 99.3 | 100.0 |
| Type VI secretion | *pppA* | HSI-I (VF0334) | 52 | 100.0 | 99.7 | 100.0 |
| Type VI secretion | *tagF/pppB* | HSI-I (VF0334) | 52 | 100.0 | 99.8 | 100.0 |
| Type VI secretion | *tagQ* | HSI-1 (Hcp-secretion island 1) (SS178) | 52 | 100.0 | 100.0 | 100.0 |
| Type VI secretion | *tagR* | HSI-I (VF0334) | 52 | 100.0 | 99.8 | 100.0 |
| Type VI secretion | *tagS* | HSI-I (VF0334) | 52 | 100.0 | 99.6 | 100.0 |
| Type VI secretion | *tagT* | HSI-I (VF0334) | 52 | 100.0 | 99.3 | 100.0 |
| Type VI secretion | *tse1* | HSI-1 (Hcp-secretion island 1) (SS178) | 52 | 100.0 | 99.8 | 100.0 |
| Type VI secretion | *tse3* | HSI-1 (Hcp-secretion island 1) (SS178) | 52 | 100.0 | 99.7 | 100.0 |
| Type VI secretion | *vgrG1a* | HSI-I (VF0334) | 52 | 100.0 | 99.8 | 100.0 |
| Type VI secretion | *vgrG1b* | HSI-1 (Hcp-secretion island 1) (SS178) | 52 | 100.0 | 99.2 | 100.0 |
| Type VI secretion | *tse2* | HSI-1 (Hcp-secretion island 1) (SS178) | 51 | 98.1 | 99.6 | 100.0 |

Supplementary Table S3. Complete numerical output for aggregate collection-year comparisons of total virulence-gene burden and virulence-module completeness.

| **Feature** | **2010 median** | **2010 IQR** | **2017 median** | **2017 IQR** | **P** | **q** |
| --- | --- | --- | --- | --- | --- | --- |
| Total virulence-gene count | 228 | 227.0–232.0 | 232 | 226.0–233.0 | 0.5955 | 0.9676 |
| Alginate and biofilm matrix | 1 | 1.000–1.000 | 1 | 1.000–1.000 | 1.0000 | 1.0000 |
| Type IV pili and adhesion | 0.9697 | 0.939–0.970 | 0.9394 | 0.939–0.970 | 0.2574 | 0.7236 |
| Flagellar motility | 0.8696 | 0.870–1.000 | 0.8696 | 0.870–1.000 | 0.7509 | 0.9761 |
| Lipopolysaccharide | 0.7143 | 0.714–0.714 | 0.7143 | 0.714–1.000 | 0.0727 | 0.7236 |
| Iron acquisition | 0.8485 | 0.848–0.970 | 0.8485 | 0.848–1.000 | 0.4712 | 0.9676 |
| Phenazine and pyocyanin | 1 | 1.000–1.000 | 1 | 1.000–1.000 | 0.2783 | 0.7236 |
| Quorum sensing and rhamnolipid | 1 | 1.000–1.000 | 1 | 1.000–1.000 | 0.7152 | 0.9761 |
| Type II secretion | 1 | 1.000–1.000 | 1 | 1.000–1.000 | 1.0000 | 1.0000 |
| Type III secretion apparatus | 1 | 1.000–1.000 | 1 | 1.000–1.000 | 1.0000 | 1.0000 |
| Type III secretion effectors | 0.75 | 0.750–0.750 | 0.75 | 0.750–0.750 | 0.5382 | 0.9676 |
| Type VI secretion | 1 | 1.000–1.000 | 1 | 1.000–1.000 | 0.2783 | 0.7236 |
| Secreted toxins and enzymes | 1 | 1.000–1.000 | 1 | 1.000–1.000 | 0.1150 | 0.7236 |

Supplementary Table S4. Gene-level comparisons of virulence-gene prevalence between the 2010 and 2017 high-quality isolate sets.

| **Gene** | **Module** | **2010 n/N** | **2017 n/N** | **P** | **q** |
| --- | --- | --- | --- | --- | --- |
| *exoS* | Type III secretion effectors | 29/29 | 20/23 | 0.0801 | 1.0000 |
| *wzy* | Lipopolysaccharide | 3/29 | 7/23 | 0.0866 | 1.0000 |
| *wzz* | Lipopolysaccharide | 3/29 | 7/23 | 0.0866 | 1.0000 |
| *pilC* | Type IV pili and adhesion | 15/29 | 8/23 | 0.2689 | 1.0000 |
| *exoU* | Type III secretion effectors | 1/29 | 3/23 | 0.3101 | 1.0000 |
| *phzH* | Phenazine and pyocyanin | 29/29 | 22/23 | 0.4423 | 1.0000 |
| *pilQ* | Type IV pili and adhesion | 29/29 | 22/23 | 0.4423 | 1.0000 |
| *plcH* | Secreted toxins and enzymes | 29/29 | 22/23 | 0.4423 | 1.0000 |
| *rhlI* | Quorum sensing and rhamnolipid | 29/29 | 22/23 | 0.4423 | 1.0000 |
| *toxA* | Secreted toxins and enzymes | 29/29 | 22/23 | 0.4423 | 1.0000 |
| *tse2* | Type VI secretion | 29/29 | 22/23 | 0.4423 | 1.0000 |
| *lasI* | Quorum sensing and rhamnolipid | 27/29 | 23/23 | 0.4970 | 1.0000 |
| *pvdJ* | Iron acquisition | 7/29 | 8/23 | 0.5396 | 1.0000 |
| *pilE* | Type IV pili and adhesion | 26/29 | 19/23 | 0.6856 | 1.0000 |
| *pilX* | Type IV pili and adhesion | 26/29 | 19/23 | 0.6856 | 1.0000 |
| *pilY1* | Type IV pili and adhesion | 26/29 | 19/23 | 0.6856 | 1.0000 |
| *pilY2* | Type IV pili and adhesion | 26/29 | 19/23 | 0.6856 | 1.0000 |
| *fpvA* | Iron acquisition | 8/29 | 8/23 | 0.7631 | 1.0000 |
| *pvdD* | Iron acquisition | 8/29 | 8/23 | 0.7631 | 1.0000 |
| *pvdE* | Iron acquisition | 8/29 | 8/23 | 0.7631 | 1.0000 |
| *pvdI* | Iron acquisition | 8/29 | 8/23 | 0.7631 | 1.0000 |
| *fleI/flag* | Flagellar motility | 12/29 | 11/23 | 0.7800 | 1.0000 |
| *fleP* | Flagellar motility | 12/29 | 11/23 | 0.7800 | 1.0000 |
| *flgL* | Flagellar motility | 12/29 | 11/23 | 0.7800 | 1.0000 |
| *fliD* | Flagellar motility | 12/29 | 11/23 | 0.7800 | 1.0000 |
| *fliS* | Flagellar motility | 12/29 | 11/23 | 0.7800 | 1.0000 |
| *alg44* | Alginate and biofilm matrix | 29/29 | 23/23 | 1.0000 | 1.0000 |
| *alg8* | Alginate and biofilm matrix | 29/29 | 23/23 | 1.0000 | 1.0000 |
| *algA* | Alginate and biofilm matrix | 29/29 | 23/23 | 1.0000 | 1.0000 |
| *algB* | Alginate and biofilm matrix | 29/29 | 23/23 | 1.0000 | 1.0000 |
| *algC* | Alginate and biofilm matrix | 29/29 | 23/23 | 1.0000 | 1.0000 |
| *algD* | Alginate and biofilm matrix | 29/29 | 23/23 | 1.0000 | 1.0000 |
| *algE* | Alginate and biofilm matrix | 29/29 | 23/23 | 1.0000 | 1.0000 |
| *algF* | Alginate and biofilm matrix | 29/29 | 23/23 | 1.0000 | 1.0000 |
| *algG* | Alginate and biofilm matrix | 29/29 | 23/23 | 1.0000 | 1.0000 |
| *algI* | Alginate and biofilm matrix | 29/29 | 23/23 | 1.0000 | 1.0000 |
| *algJ* | Alginate and biofilm matrix | 29/29 | 23/23 | 1.0000 | 1.0000 |
| *algK* | Alginate and biofilm matrix | 29/29 | 23/23 | 1.0000 | 1.0000 |
| *algL* | Alginate and biofilm matrix | 29/29 | 23/23 | 1.0000 | 1.0000 |
| *algP/algR3* | Alginate and biofilm matrix | 29/29 | 23/23 | 1.0000 | 1.0000 |
| *algQ* | Alginate and biofilm matrix | 29/29 | 23/23 | 1.0000 | 1.0000 |
| *algR* | Alginate and biofilm matrix | 29/29 | 23/23 | 1.0000 | 1.0000 |
| *algU* | Alginate and biofilm matrix | 29/29 | 23/23 | 1.0000 | 1.0000 |
| *algW* | Alginate and biofilm matrix | 29/29 | 23/23 | 1.0000 | 1.0000 |
| *algX* | Alginate and biofilm matrix | 29/29 | 23/23 | 1.0000 | 1.0000 |
| *algZ* | Alginate and biofilm matrix | 29/29 | 23/23 | 1.0000 | 1.0000 |
| *aprA* | Secreted toxins and enzymes | 29/29 | 23/23 | 1.0000 | 1.0000 |
| *chpA* | Type IV pili and adhesion | 29/29 | 23/23 | 1.0000 | 1.0000 |
| *chpB* | Type IV pili and adhesion | 29/29 | 23/23 | 1.0000 | 1.0000 |
| *chpC* | Type IV pili and adhesion | 29/29 | 23/23 | 1.0000 | 1.0000 |
| *chpD* | Type IV pili and adhesion | 29/29 | 23/23 | 1.0000 | 1.0000 |
| *chpE* | Type IV pili and adhesion | 29/29 | 23/23 | 1.0000 | 1.0000 |
| *clpV1* | Type VI secretion | 29/29 | 23/23 | 1.0000 | 1.0000 |
| *dotU1* | Type VI secretion | 29/29 | 23/23 | 1.0000 | 1.0000 |
| *exoT* | Type III secretion effectors | 29/29 | 23/23 | 1.0000 | 1.0000 |
| *exoY* | Type III secretion effectors | 28/29 | 22/23 | 1.0000 | 1.0000 |
| *exsA* | Type III secretion apparatus | 29/29 | 23/23 | 1.0000 | 1.0000 |
| *exsB* | Type III secretion apparatus | 29/29 | 23/23 | 1.0000 | 1.0000 |
| *exsC* | Type III secretion apparatus | 29/29 | 23/23 | 1.0000 | 1.0000 |
| *exsD* | Type III secretion apparatus | 29/29 | 23/23 | 1.0000 | 1.0000 |
| *exsE* | Type III secretion apparatus | 29/29 | 23/23 | 1.0000 | 1.0000 |
| *fha1* | Type VI secretion | 29/29 | 23/23 | 1.0000 | 1.0000 |
| *fimT* | Type IV pili and adhesion | 25/29 | 19/23 | 1.0000 | 1.0000 |
| *fimU* | Type IV pili and adhesion | 25/29 | 19/23 | 1.0000 | 1.0000 |
| *fimV* | Type IV pili and adhesion | 29/29 | 23/23 | 1.0000 | 1.0000 |
| *fleN* | Flagellar motility | 29/29 | 23/23 | 1.0000 | 1.0000 |
| *fleQ* | Flagellar motility | 29/29 | 23/23 | 1.0000 | 1.0000 |
| *fleR* | Flagellar motility | 29/29 | 23/23 | 1.0000 | 1.0000 |
| *fleS* | Flagellar motility | 29/29 | 23/23 | 1.0000 | 1.0000 |
| *flgA* | Flagellar motility | 29/29 | 23/23 | 1.0000 | 1.0000 |
| *flgB* | Flagellar motility | 29/29 | 23/23 | 1.0000 | 1.0000 |
| *flgC* | Flagellar motility | 29/29 | 23/23 | 1.0000 | 1.0000 |
| *flgD* | Flagellar motility | 29/29 | 23/23 | 1.0000 | 1.0000 |
| *flgE* | Flagellar motility | 29/29 | 23/23 | 1.0000 | 1.0000 |
| *flgF* | Flagellar motility | 29/29 | 23/23 | 1.0000 | 1.0000 |
| *flgG* | Flagellar motility | 29/29 | 23/23 | 1.0000 | 1.0000 |
| *flgH* | Flagellar motility | 29/29 | 23/23 | 1.0000 | 1.0000 |
| *flgI* | Flagellar motility | 29/29 | 23/23 | 1.0000 | 1.0000 |
| *flgJ* | Flagellar motility | 29/29 | 23/23 | 1.0000 | 1.0000 |
| *flgK* | Flagellar motility | 29/29 | 23/23 | 1.0000 | 1.0000 |
| *flgM* | Flagellar motility | 29/29 | 23/23 | 1.0000 | 1.0000 |
| *flgN* | Flagellar motility | 29/29 | 23/23 | 1.0000 | 1.0000 |
| *flhA* | Flagellar motility | 29/29 | 23/23 | 1.0000 | 1.0000 |
| *flhB* | Flagellar motility | 29/29 | 23/23 | 1.0000 | 1.0000 |
| *flhF* | Flagellar motility | 29/29 | 23/23 | 1.0000 | 1.0000 |
| *fliA* | Flagellar motility | 29/29 | 23/23 | 1.0000 | 1.0000 |
| *fliC* | Flagellar motility | 13/29 | 11/23 | 1.0000 | 1.0000 |
| *fliE* | Flagellar motility | 29/29 | 23/23 | 1.0000 | 1.0000 |
| *fliF* | Flagellar motility | 29/29 | 23/23 | 1.0000 | 1.0000 |
| *fliG* | Flagellar motility | 29/29 | 23/23 | 1.0000 | 1.0000 |
| *fliH* | Flagellar motility | 29/29 | 23/23 | 1.0000 | 1.0000 |
| *fliI* | Flagellar motility | 29/29 | 23/23 | 1.0000 | 1.0000 |
| *fliJ* | Flagellar motility | 29/29 | 23/23 | 1.0000 | 1.0000 |
| *fliK* | Flagellar motility | 29/29 | 23/23 | 1.0000 | 1.0000 |
| *fliL* | Flagellar motility | 29/29 | 23/23 | 1.0000 | 1.0000 |
| *fliM* | Flagellar motility | 29/29 | 23/23 | 1.0000 | 1.0000 |
| *fliN* | Flagellar motility | 29/29 | 23/23 | 1.0000 | 1.0000 |
| *fliO* | Flagellar motility | 29/29 | 23/23 | 1.0000 | 1.0000 |
| *fliP* | Flagellar motility | 29/29 | 23/23 | 1.0000 | 1.0000 |
| *fliQ* | Flagellar motility | 29/29 | 23/23 | 1.0000 | 1.0000 |
| *fliR* | Flagellar motility | 29/29 | 23/23 | 1.0000 | 1.0000 |
| *fptA* | Iron acquisition | 29/29 | 23/23 | 1.0000 | 1.0000 |
| *hcp1* | Type VI secretion | 29/29 | 23/23 | 1.0000 | 1.0000 |
| *hsiA1* | Type VI secretion | 29/29 | 23/23 | 1.0000 | 1.0000 |
| *hsiB1/vipA* | Type VI secretion | 29/29 | 23/23 | 1.0000 | 1.0000 |
| *hsiC1/vipB* | Type VI secretion | 29/29 | 23/23 | 1.0000 | 1.0000 |
| *hsiE1* | Type VI secretion | 29/29 | 23/23 | 1.0000 | 1.0000 |
| *hsiF1* | Type VI secretion | 29/29 | 23/23 | 1.0000 | 1.0000 |
| *hsiG1* | Type VI secretion | 29/29 | 23/23 | 1.0000 | 1.0000 |
| *hsiH1* | Type VI secretion | 29/29 | 23/23 | 1.0000 | 1.0000 |
| *hsiJ1* | Type VI secretion | 29/29 | 23/23 | 1.0000 | 1.0000 |
| *icmF1/tssM1* | Type VI secretion | 29/29 | 23/23 | 1.0000 | 1.0000 |
| *lasA* | Secreted toxins and enzymes | 29/29 | 23/23 | 1.0000 | 1.0000 |
| *lasB* | Secreted toxins and enzymes | 29/29 | 23/23 | 1.0000 | 1.0000 |
| *lip1* | Type VI secretion | 29/29 | 23/23 | 1.0000 | 1.0000 |
| *mbtH-like* | Iron acquisition | 29/29 | 23/23 | 1.0000 | 1.0000 |
| *motA* | Flagellar motility | 29/29 | 23/23 | 1.0000 | 1.0000 |
| *motB* | Flagellar motility | 29/29 | 23/23 | 1.0000 | 1.0000 |
| *motC* | Flagellar motility | 29/29 | 23/23 | 1.0000 | 1.0000 |
| *motD* | Flagellar motility | 29/29 | 23/23 | 1.0000 | 1.0000 |
| *motY* | Flagellar motility | 29/29 | 23/23 | 1.0000 | 1.0000 |
| *mucA* | Alginate and biofilm matrix | 29/29 | 23/23 | 1.0000 | 1.0000 |
| *mucB* | Alginate and biofilm matrix | 29/29 | 23/23 | 1.0000 | 1.0000 |
| *mucC* | Alginate and biofilm matrix | 29/29 | 23/23 | 1.0000 | 1.0000 |
| *mucD* | Alginate and biofilm matrix | 29/29 | 23/23 | 1.0000 | 1.0000 |
| *mucE* | Alginate and biofilm matrix | 29/29 | 23/23 | 1.0000 | 1.0000 |
| *mucP* | Alginate and biofilm matrix | 29/29 | 23/23 | 1.0000 | 1.0000 |
| *pchA* | Iron acquisition | 29/29 | 23/23 | 1.0000 | 1.0000 |
| *pchB* | Iron acquisition | 29/29 | 23/23 | 1.0000 | 1.0000 |
| *pchC* | Iron acquisition | 29/29 | 23/23 | 1.0000 | 1.0000 |
| *pchD* | Iron acquisition | 29/29 | 23/23 | 1.0000 | 1.0000 |
| *pchE* | Iron acquisition | 28/29 | 23/23 | 1.0000 | 1.0000 |
| *pchF* | Iron acquisition | 29/29 | 23/23 | 1.0000 | 1.0000 |
| *pchG* | Iron acquisition | 29/29 | 23/23 | 1.0000 | 1.0000 |
| *pchH* | Iron acquisition | 29/29 | 23/23 | 1.0000 | 1.0000 |
| *pchI* | Iron acquisition | 29/29 | 23/23 | 1.0000 | 1.0000 |
| *pchR* | Iron acquisition | 29/29 | 23/23 | 1.0000 | 1.0000 |
| *pcr1* | Type III secretion apparatus | 29/29 | 23/23 | 1.0000 | 1.0000 |
| *pcr2* | Type III secretion apparatus | 29/29 | 23/23 | 1.0000 | 1.0000 |
| *pcr3* | Type III secretion apparatus | 29/29 | 23/23 | 1.0000 | 1.0000 |
| *pcr4* | Type III secretion apparatus | 29/29 | 23/23 | 1.0000 | 1.0000 |
| *pcrD* | Type III secretion apparatus | 29/29 | 23/23 | 1.0000 | 1.0000 |
| *pcrG* | Type III secretion apparatus | 29/29 | 23/23 | 1.0000 | 1.0000 |
| *pcrH* | Type III secretion apparatus | 29/29 | 23/23 | 1.0000 | 1.0000 |
| *pcrR* | Type III secretion apparatus | 29/29 | 23/23 | 1.0000 | 1.0000 |
| *pcrV* | Type III secretion apparatus | 29/29 | 23/23 | 1.0000 | 1.0000 |
| *phzA1* | Phenazine and pyocyanin | 29/29 | 23/23 | 1.0000 | 1.0000 |
| *phzB1* | Phenazine and pyocyanin | 29/29 | 23/23 | 1.0000 | 1.0000 |
| *phzC1* | Phenazine and pyocyanin | 29/29 | 23/23 | 1.0000 | 1.0000 |
| *phzD1* | Phenazine and pyocyanin | 29/29 | 23/23 | 1.0000 | 1.0000 |
| *phzE1* | Phenazine and pyocyanin | 29/29 | 23/23 | 1.0000 | 1.0000 |
| *phzF1* | Phenazine and pyocyanin | 29/29 | 23/23 | 1.0000 | 1.0000 |
| *phzG1* | Phenazine and pyocyanin | 29/29 | 23/23 | 1.0000 | 1.0000 |
| *phzM* | Phenazine and pyocyanin | 29/29 | 23/23 | 1.0000 | 1.0000 |
| *phzS* | Phenazine and pyocyanin | 29/29 | 23/23 | 1.0000 | 1.0000 |
| *pilA* | Type IV pili and adhesion | 1/29 | 1/23 | 1.0000 | 1.0000 |
| *pilB* | Type IV pili and adhesion | 29/29 | 23/23 | 1.0000 | 1.0000 |
| *pilF* | Type IV pili and adhesion | 29/29 | 23/23 | 1.0000 | 1.0000 |
| *pilG* | Type IV pili and adhesion | 29/29 | 23/23 | 1.0000 | 1.0000 |
| *pilH* | Type IV pili and adhesion | 29/29 | 23/23 | 1.0000 | 1.0000 |
| *pilI* | Type IV pili and adhesion | 29/29 | 23/23 | 1.0000 | 1.0000 |
| *pilJ* | Type IV pili and adhesion | 29/29 | 23/23 | 1.0000 | 1.0000 |
| *pilK* | Type IV pili and adhesion | 29/29 | 23/23 | 1.0000 | 1.0000 |
| *pilM* | Type IV pili and adhesion | 29/29 | 23/23 | 1.0000 | 1.0000 |
| *pilN* | Type IV pili and adhesion | 29/29 | 23/23 | 1.0000 | 1.0000 |
| *pilO* | Type IV pili and adhesion | 29/29 | 23/23 | 1.0000 | 1.0000 |
| *pilP* | Type IV pili and adhesion | 29/29 | 23/23 | 1.0000 | 1.0000 |
| *pilR* | Type IV pili and adhesion | 29/29 | 23/23 | 1.0000 | 1.0000 |
| *pilS* | Type IV pili and adhesion | 29/29 | 23/23 | 1.0000 | 1.0000 |
| *pilT* | Type IV pili and adhesion | 29/29 | 23/23 | 1.0000 | 1.0000 |
| *pilU* | Type IV pili and adhesion | 29/29 | 23/23 | 1.0000 | 1.0000 |
| *pilV* | Type IV pili and adhesion | 25/29 | 19/23 | 1.0000 | 1.0000 |
| *pilW* | Type IV pili and adhesion | 25/29 | 19/23 | 1.0000 | 1.0000 |
| *popB* | Type III secretion apparatus | 29/29 | 23/23 | 1.0000 | 1.0000 |
| *popD* | Type III secretion apparatus | 29/29 | 23/23 | 1.0000 | 1.0000 |
| *popN* | Type III secretion apparatus | 29/29 | 23/23 | 1.0000 | 1.0000 |
| *ppkA* | Type VI secretion | 29/29 | 23/23 | 1.0000 | 1.0000 |
| *pppA* | Type VI secretion | 29/29 | 23/23 | 1.0000 | 1.0000 |
| *pscB* | Type III secretion apparatus | 29/29 | 23/23 | 1.0000 | 1.0000 |
| *pscC* | Type III secretion apparatus | 29/29 | 23/23 | 1.0000 | 1.0000 |
| *pscD* | Type III secretion apparatus | 29/29 | 23/23 | 1.0000 | 1.0000 |
| *pscE* | Type III secretion apparatus | 29/29 | 23/23 | 1.0000 | 1.0000 |
| *pscF* | Type III secretion apparatus | 29/29 | 23/23 | 1.0000 | 1.0000 |
| *pscG* | Type III secretion apparatus | 29/29 | 23/23 | 1.0000 | 1.0000 |
| *pscH* | Type III secretion apparatus | 29/29 | 23/23 | 1.0000 | 1.0000 |
| *pscI* | Type III secretion apparatus | 29/29 | 23/23 | 1.0000 | 1.0000 |
| *pscJ* | Type III secretion apparatus | 29/29 | 23/23 | 1.0000 | 1.0000 |
| *pscK* | Type III secretion apparatus | 29/29 | 23/23 | 1.0000 | 1.0000 |
| *pscL* | Type III secretion apparatus | 29/29 | 23/23 | 1.0000 | 1.0000 |
| *pscN* | Type III secretion apparatus | 29/29 | 23/23 | 1.0000 | 1.0000 |
| *pscO* | Type III secretion apparatus | 29/29 | 23/23 | 1.0000 | 1.0000 |
| *pscP* | Type III secretion apparatus | 29/29 | 23/23 | 1.0000 | 1.0000 |
| *pscQ* | Type III secretion apparatus | 29/29 | 23/23 | 1.0000 | 1.0000 |
| *pscR* | Type III secretion apparatus | 29/29 | 23/23 | 1.0000 | 1.0000 |
| *pscS* | Type III secretion apparatus | 29/29 | 23/23 | 1.0000 | 1.0000 |
| *pscT* | Type III secretion apparatus | 29/29 | 23/23 | 1.0000 | 1.0000 |
| *pscU* | Type III secretion apparatus | 29/29 | 23/23 | 1.0000 | 1.0000 |
| *ptxR* | Iron acquisition | 29/29 | 23/23 | 1.0000 | 1.0000 |
| *pvcA* | Iron acquisition | 29/29 | 23/23 | 1.0000 | 1.0000 |
| *pvcB* | Iron acquisition | 29/29 | 23/23 | 1.0000 | 1.0000 |
| *pvcC* | Iron acquisition | 29/29 | 23/23 | 1.0000 | 1.0000 |
| *pvcD* | Iron acquisition | 29/29 | 23/23 | 1.0000 | 1.0000 |
| *pvdA* | Iron acquisition | 29/29 | 23/23 | 1.0000 | 1.0000 |
| *pvdF* | Iron acquisition | 29/29 | 23/23 | 1.0000 | 1.0000 |
| *pvdG* | Iron acquisition | 29/29 | 23/23 | 1.0000 | 1.0000 |
| *pvdH* | Iron acquisition | 29/29 | 23/23 | 1.0000 | 1.0000 |
| *pvdL* | Iron acquisition | 29/29 | 23/23 | 1.0000 | 1.0000 |
| *pvdM* | Iron acquisition | 29/29 | 23/23 | 1.0000 | 1.0000 |
| *pvdN* | Iron acquisition | 29/29 | 23/23 | 1.0000 | 1.0000 |
| *pvdO* | Iron acquisition | 29/29 | 23/23 | 1.0000 | 1.0000 |
| *pvdP* | Iron acquisition | 29/29 | 23/23 | 1.0000 | 1.0000 |
| *pvdQ* | Iron acquisition | 29/29 | 23/23 | 1.0000 | 1.0000 |
| *pvdS* | Iron acquisition | 29/29 | 23/23 | 1.0000 | 1.0000 |
| *rhlA* | Quorum sensing and rhamnolipid | 29/29 | 23/23 | 1.0000 | 1.0000 |
| *rhlB* | Quorum sensing and rhamnolipid | 29/29 | 23/23 | 1.0000 | 1.0000 |
| *rhlC* | Quorum sensing and rhamnolipid | 29/29 | 23/23 | 1.0000 | 1.0000 |
| *tagF/pppB* | Type VI secretion | 29/29 | 23/23 | 1.0000 | 1.0000 |
| *tagQ* | Type VI secretion | 29/29 | 23/23 | 1.0000 | 1.0000 |
| *tagR* | Type VI secretion | 29/29 | 23/23 | 1.0000 | 1.0000 |
| *tagS* | Type VI secretion | 29/29 | 23/23 | 1.0000 | 1.0000 |
| *tagT* | Type VI secretion | 29/29 | 23/23 | 1.0000 | 1.0000 |
| *tse1* | Type VI secretion | 29/29 | 23/23 | 1.0000 | 1.0000 |
| *tse3* | Type VI secretion | 29/29 | 23/23 | 1.0000 | 1.0000 |
| *vgrG1a* | Type VI secretion | 29/29 | 23/23 | 1.0000 | 1.0000 |
| *vgrG1b* | Type VI secretion | 29/29 | 23/23 | 1.0000 | 1.0000 |
| *waaA* | Lipopolysaccharide | 29/29 | 23/23 | 1.0000 | 1.0000 |
| *waaC* | Lipopolysaccharide | 29/29 | 23/23 | 1.0000 | 1.0000 |
| *waaF* | Lipopolysaccharide | 29/29 | 23/23 | 1.0000 | 1.0000 |
| *waaG* | Lipopolysaccharide | 29/29 | 23/23 | 1.0000 | 1.0000 |
| *waaP* | Lipopolysaccharide | 29/29 | 23/23 | 1.0000 | 1.0000 |
| *xcpA/pilD* | Type IV pili and adhesion | 29/29 | 23/23 | 1.0000 | 1.0000 |
| *xcpP* | Type II secretion | 29/29 | 23/23 | 1.0000 | 1.0000 |
| *xcpQ* | Type II secretion | 29/29 | 23/23 | 1.0000 | 1.0000 |
| *xcpR* | Type II secretion | 29/29 | 23/23 | 1.0000 | 1.0000 |
| *xcpS* | Type II secretion | 29/29 | 23/23 | 1.0000 | 1.0000 |
| *xcpT* | Type II secretion | 29/29 | 23/23 | 1.0000 | 1.0000 |
| *xcpU* | Type II secretion | 29/29 | 23/23 | 1.0000 | 1.0000 |
| *xcpV* | Type II secretion | 29/29 | 23/23 | 1.0000 | 1.0000 |
| *xcpW* | Type II secretion | 29/29 | 23/23 | 1.0000 | 1.0000 |
| *xcpX* | Type II secretion | 29/29 | 23/23 | 1.0000 | 1.0000 |
| *xcpY* | Type II secretion | 29/29 | 23/23 | 1.0000 | 1.0000 |
| *xcpZ* | Type II secretion | 29/29 | 23/23 | 1.0000 | 1.0000 |

Supplementary Table S5. Sensitivity of selected virulence-gene prevalence estimates to the primary 80% identity/80% coverage and strict 90% identity/90% coverage VFDB thresholds.

| **Gene** | **Module** | **80/80 n (%)** | **90/90 n (%)** |
| --- | --- | --- | --- |
| *algD* | Alginate and biofilm matrix | 52 (100.0) | 52 (100.0) |
| *alg8* | Alginate and biofilm matrix | 52 (100.0) | 52 (100.0) |
| *alg44* | Alginate and biofilm matrix | 52 (100.0) | 52 (100.0) |
| *pilA* | Type IV pili and adhesion | 2 (3.8) | 1 (1.9) |
| *pilB* | Type IV pili and adhesion | 52 (100.0) | 13 (25.0) |
| *pilT* | Type IV pili and adhesion | 52 (100.0) | 52 (100.0) |
| *fimV* | Type IV pili and adhesion | 52 (100.0) | 52 (100.0) |
| *fliC* | Flagellar motility | 24 (46.2) | 24 (46.2) |
| *fleQ* | Flagellar motility | 52 (100.0) | 52 (100.0) |
| *pvdS* | Iron acquisition | 52 (100.0) | 52 (100.0) |
| *pvdA* | Iron acquisition | 52 (100.0) | 30 (57.7) |
| *pvdD* | Iron acquisition | 16 (30.8) | 16 (30.8) |
| *pvdE* | Iron acquisition | 16 (30.8) | 16 (30.8) |
| *fpvA* | Iron acquisition | 16 (30.8) | 16 (30.8) |
| *pchE* | Iron acquisition | 51 (98.1) | 51 (98.1) |
| *pchF* | Iron acquisition | 52 (100.0) | 52 (100.0) |
| *lasI* | Quorum sensing and rhamnolipid | 50 (96.2) | 50 (96.2) |
| *rhlI* | Quorum sensing and rhamnolipid | 51 (98.1) | 51 (98.1) |
| *rhlA* | Quorum sensing and rhamnolipid | 52 (100.0) | 52 (100.0) |
| *rhlB* | Quorum sensing and rhamnolipid | 52 (100.0) | 52 (100.0) |
| *phzM* | Phenazine and pyocyanin | 52 (100.0) | 52 (100.0) |
| *phzS* | Phenazine and pyocyanin | 52 (100.0) | 52 (100.0) |
| *toxA* | Secreted toxins and enzymes | 51 (98.1) | 51 (98.1) |
| *lasA* | Secreted toxins and enzymes | 52 (100.0) | 52 (100.0) |
| *lasB* | Secreted toxins and enzymes | 52 (100.0) | 52 (100.0) |
| *aprA* | Secreted toxins and enzymes | 52 (100.0) | 52 (100.0) |
| *plcH* | Secreted toxins and enzymes | 51 (98.1) | 51 (98.1) |
| *exoT* | Type III secretion effectors | 52 (100.0) | 52 (100.0) |
| *exoY* | Type III secretion effectors | 50 (96.2) | 50 (96.2) |
| *exoS* | Type III secretion effectors | 49 (94.2) | 49 (94.2) |
| *exoU* | Type III secretion effectors | 4 (7.7) | 4 (7.7) |
| *pscC* | Type III secretion apparatus | 52 (100.0) | 52 (100.0) |
| *pscN* | Type III secretion apparatus | 52 (100.0) | 52 (100.0) |
| *xcpQ* | Type II secretion | 52 (100.0) | 52 (100.0) |
| *hcp1* | Type VI secretion | 52 (100.0) | 52 (100.0) |
| *icmF1/tssM1* | Type VI secretion | 52 (100.0) | 52 (100.0) |

Supplementary Table S6. Isolate-level T3SS effector profiles in the 52 high-quality genomes.

| **Isolate** | **Year** | **Principal virulotype** | **Full four-effector profile** |
| --- | --- | --- | --- |
| 71 | 2017 | *exoS+/exoU−* | *exoS+;exoU−;exoT+;exoY+* |
| 72 | 2017 | *exoS+/exoU−* | *exoS+;exoU−;exoT+;exoY+* |
| 73 | 2017 | *exoS+/exoU−* | *exoS+;exoU−;exoT+;exoY+* |
| 74 | 2017 | *exoS+/exoU−* | *exoS+;exoU−;exoT+;exoY+* |
| 75 | 2017 | *exoS+/exoU−* | *exoS+;exoU−;exoT+;exoY+* |
| 76 | 2017 | *exoS+/exoU−* | *exoS+;exoU−;exoT+;exoY+* |
| 78 | 2017 | *exoS+/exoU−* | *exoS+;exoU−;exoT+;exoY+* |
| 80 | 2017 | *exoS+/exoU−* | *exoS+;exoU−;exoT+;exoY+* |
| 81 | 2017 | *exoS+/exoU−* | *exoS+;exoU−;exoT+;exoY+* |
| 82 | 2017 | *exoS+/exoU−* | *exoS+;exoU−;exoT+;exoY+* |
| 83 | 2017 | *exoS+/exoU−* | *exoS+;exoU−;exoT+;exoY−* |
| 84 | 2017 | *exoS+/exoU−* | *exoS+;exoU−;exoT+;exoY+* |
| 85 | 2017 | *exoS+/exoU−* | *exoS+;exoU−;exoT+;exoY+* |
| 86 | 2017 | *exoU+/exoS−* | *exoS−;exoU+;exoT+;exoY+* |
| 87 | 2017 | *exoS+/exoU−* | *exoS+;exoU−;exoT+;exoY+* |
| 88 | 2010 | *exoS+/exoU−* | *exoS+;exoU−;exoT+;exoY+* |
| 89 | 2010 | *exoS+/exoU+* | *exoS+;exoU+;exoT+;exoY+* |
| 90 | 2010 | *exoS+/exoU−* | *exoS+;exoU−;exoT+;exoY+* |
| 91 | 2010 | *exoS+/exoU−* | *exoS+;exoU−;exoT+;exoY+* |
| 92 | 2010 | *exoS+/exoU−* | *exoS+;exoU−;exoT+;exoY+* |
| 93 | 2017 | *exoU+/exoS−* | *exoS−;exoU+;exoT+;exoY+* |
| 94 | 2010 | *exoS+/exoU−* | *exoS+;exoU−;exoT+;exoY+* |
| 95 | 2010 | *exoS+/exoU−* | *exoS+;exoU−;exoT+;exoY+* |
| 96 | 2010 | *exoS+/exoU−* | *exoS+;exoU−;exoT+;exoY+* |
| 97 | 2010 | *exoS+/exoU−* | *exoS+;exoU−;exoT+;exoY+* |
| 98 | 2010 | *exoS+/exoU−* | *exoS+;exoU−;exoT+;exoY−* |
| 99 | 2010 | *exoS+/exoU−* | *exoS+;exoU−;exoT+;exoY+* |
| 100 | 2010 | *exoS+/exoU−* | *exoS+;exoU−;exoT+;exoY+* |
| 101 | 2010 | *exoS+/exoU−* | *exoS+;exoU−;exoT+;exoY+* |
| 102 | 2010 | *exoS+/exoU−* | *exoS+;exoU−;exoT+;exoY+* |
| 103 | 2010 | *exoS+/exoU−* | *exoS+;exoU−;exoT+;exoY+* |
| 104 | 2010 | *exoS+/exoU−* | *exoS+;exoU−;exoT+;exoY+* |
| 105 | 2010 | *exoS+/exoU−* | *exoS+;exoU−;exoT+;exoY+* |
| 106 | 2010 | *exoS+/exoU−* | *exoS+;exoU−;exoT+;exoY+* |
| 107 | 2010 | *exoS+/exoU−* | *exoS+;exoU−;exoT+;exoY+* |
| 108 | 2010 | *exoS+/exoU−* | *exoS+;exoU−;exoT+;exoY+* |
| 109 | 2010 | *exoS+/exoU−* | *exoS+;exoU−;exoT+;exoY+* |
| 111 | 2010 | *exoS+/exoU−* | *exoS+;exoU−;exoT+;exoY+* |
| 112 | 2017 | *exoS+/exoU−* | *exoS+;exoU−;exoT+;exoY+* |
| 113 | 2017 | *exoS+/exoU−* | *exoS+;exoU−;exoT+;exoY+* |
| 114 | 2010 | *exoS+/exoU−* | *exoS+;exoU−;exoT+;exoY+* |
| 115 | 2010 | *exoS+/exoU−* | *exoS+;exoU−;exoT+;exoY+* |
| 116 | 2010 | *exoS+/exoU−* | *exoS+;exoU−;exoT+;exoY+* |
| 117 | 2010 | *exoS+/exoU−* | *exoS+;exoU−;exoT+;exoY+* |
| 118 | 2010 | *exoS+/exoU−* | *exoS+;exoU−;exoT+;exoY+* |
| 121 | 2010 | *exoS+/exoU−* | *exoS+;exoU−;exoT+;exoY+* |
| 122 | 2010 | *exoS+/exoU−* | *exoS+;exoU−;exoT+;exoY+* |
| 123 | 2017 | *exoS+/exoU−* | *exoS+;exoU−;exoT+;exoY+* |
| 124 | 2017 | *exoS+/exoU−* | *exoS+;exoU−;exoT+;exoY+* |
| 125 | 2017 | *exoS+/exoU−* | *exoS+;exoU−;exoT+;exoY+* |
| 127 | 2017 | *exoS+/exoU−* | *exoS+;exoU−;exoT+;exoY+* |
| 129 | 2017 | *exoU+/exoS−* | *exoS−;exoU+;exoT+;exoY+* |

Supplementary Figure S1. Virulence-gene prevalence spectrum. Genes are ranked by prevalence among the 52 high-quality genomes. Horizontal lines indicate the cohort-defined core-like (95%), soft-core (90%) and shell/cloud (15%) boundaries. The inset gives the number of genes in each prevalence category.

Supplementary Figure S2. Sensitivity of selected-gene prevalence to VFDB thresholds. Points compare prevalence under the primary 80% identity/80% coverage criterion and a stricter 90%/90% criterion. Large threshold-associated changes for selected loci emphasize allele-dependent reference matching.

Supplementary Figure S3. Virulence-module completeness. Each cell shows the proportion of the cohort-defined union of genes in a functional module detected in an isolate. Isolate order matches Figure 3 of the main manuscript. Module completeness describes reference-gene detection and is not a direct functional assay.

Supplementary Figure S4. Descriptive core-genome maximum-likelihood topology of the 59 historical genome-confirmed *Pseudomonas aeruginosa* assemblies. The topology was derived from a Panaroo core-gene alignment using IQ-TREE v3.0.1 under the GTR+F+I+R5 nucleotide-substitution model selected by ModelFinder. Annotation tracks indicate collection year, genome-quality status, principal T3SS genomic profile and selected virulence-gene calls. No bootstrap or SH-aLRT resampling was performed in the original analysis; consequently, the topology is provided only as supplementary descriptive documentation and is not interpreted as evidence of supported clades, lineage relationships, temporal population structure, cross-host relationships or transmission.
